# GEC-DTSP: A GNN–RL-based Edge–Cloud Digital Twin framework for real-time traffic forecasting and adaptive signal control

**DOI:** 10.1371/journal.pone.0350247

**Published:** 2026-06-01

**Authors:** Fayez Alanazi, Ammar Armghan, Ahmed Jamal Abdullah Al-Gburi, Amr Yousef

**Affiliations:** 1 Civil Engineering Department, College of Engineering, Jouf University, Sakaka, Saudi Arabia; 2 Department of Electrical Engineering, College of Engineering, Jouf University, Sakaka, Saudi Arabia; 3 Strategic Research Institute (SRI), Asia Pacific University, Jalan Teknologi 5, Taman Teknologi Malaysia, Kuala Lumpur, Malaysia; 4 Department of Electrical Engineering, College of Engineering, University of Business and Technology, Jeddah, Saudi Arabia; 5 Engineering Mathematics Department, Faculty of Engineering, Alexandria University, Alexandria, Egypt; National University of Defense Technology, CHINA

## Abstract

Urban traffic networks exhibit highly dynamic and nonlinear spatiotemporal interactions that require predictive modeling and adaptive control mechanisms capable of operating under low-latency constraints. This study proposes a GNN–RL-based Edge–Cloud Digital Twin framework for real-time traffic forecasting and adaptive signal control. At the network edge, multi-source traffic data collected from roadside sensors are processed on distributed edge devices to perform multi-step prediction of traffic flow, vehicle density, and congestion states. The forecasting module integrates Graph Convolutional Networks (GCNs) to capture spatial dependencies across the road topology with Long Short-Term Memory (LSTM) units and a Transformer-based predictor to model short- and long-range temporal dynamics. These predicted traffic states are transmitted to a cloud-level Digital Twin Engine, which performs data fusion, state estimation, calibration, and scenario-based simulation to maintain a continuously updated virtual representation of the physical traffic network. Using the forecasted states as inputs, a deep reinforcement learning optimization module performs adaptive signal phase control to minimize average vehicle delay and maximize intersection throughput. The overall framework operates as a closed feedback loop integrating edge-level spatiotemporal forecasting, cloud-level synchronization and simulation, and reinforcement learning–based control policy optimization. Experimental evaluation demonstrates a 17% reduction in average vehicle waiting time and significant improvements in forecasting performance measured using MAE and RMSE, with strong robustness to missing and noisy data conditions. The proposed architecture provides a scalable and low-latency solution for data-driven traffic prediction and signal control within an edge–cloud digital twin environment.

## 1. Introduction

### 1.1 Background

Digital Twin (DT) technology has emerged as a transformative paradigm in intelligent transportation systems, offering a virtual representation of real-world infrastructure for real-time monitoring, prediction, and decision-making [1]. Edge–cloud computing integrates the low-latency, real-time processing power of edge devices with the scalability and storage capabilities of cloud servers for efficient data management [2]. By synchronizing physical traffic networks with computational models, DT enables continuous feedback loops that support operational efficiency and resilience [3]. Modern cities increasingly adopt DT frameworks to simulate congestion, evaluate policies, and enhance transport safety. The integration of Internet of Things (IoT) devices has further accelerated DT applications, as vast sensor-generated data streams provide the necessary input for realistic simulations [4]. However, despite advancements, most DT traffic platforms rely on centralized processing, limiting responsiveness under heavy loads. Addressing these bottlenecks is crucial for building scalable and adaptive traffic ecosystems [5].

Digitalization of urban mobility introduces immense opportunities but also presents several challenges. Traditional systems, constrained by fixed-timing signal control, exhibit limited adaptability under dynamic conditions [6]. The sheer volume of heterogeneous traffic data from vehicles, sensors, and cameras increases computational demands, making centralized processing insufficient [7]. Moreover, current predictive models often fail to capture nonlinear spatiotemporal patterns, resulting in suboptimal forecasts. These issues hinder proactive congestion management, raising concerns about sustainability and safety [8]. Thus, a shift toward distributed, intelligent, and cloud-integrated Digital Twin platforms is essential to meet the growing demands of urban mobility [9].

Edge-cloud integration represents a promising architecture for real-time traffic intelligence, distributing processing between local edge devices and centralized cloud servers [10]. Edge nodes located near sensors enable low-latency decision-making, crucial for dynamic tasks such as adaptive traffic signal control [11]. Simultaneously, cloud servers host advanced analytics and long-term strategic models, aggregating historical and city-wide traffic data to optimize the system [12]. This hybrid architecture supports scalability while maintaining responsiveness, positioning edge-cloud synergy as a central enabler of modern traffic management systems. Such architectures also reduce bandwidth load, since only relevant data is sent to the cloud, minimizing redundancy and improving efficiency [13].

The application of edge-cloud models to traffic systems aligns with broader smart city initiatives. By leveraging edge computing for immediate responses and cloud computing for large-scale predictive modeling, cities can achieve both agility and strategic planning [14]. However, challenges remain in ensuring synchronization between edge and cloud layers, securing data privacy, and managing heterogeneous infrastructures [15]. Furthermore, integrating machine learning and reinforcement learning algorithms within edge-cloud platforms requires robust orchestration frameworks [16]. These challenges underscore the need for carefully designed architectures that can deliver accuracy, scalability, and resilience in traffic operations.

### 1.2 Challenges

Urban traffic systems are becoming increasingly complex due to rapid urbanization, rising vehicle ownership, and growing demand for sustainable mobility. Traditional traffic management approaches, which rely on centralized processing and static control strategies, struggle to adapt under dynamic conditions. These systems suffer from high latency, limited predictive capabilities, and poor scalability, resulting in congestion, delays, and inefficiency. Furthermore, current predictive models often struggle to capture complex, nonlinear spatial and temporal relationships within road networks, thereby limiting the accuracy of traffic forecasts. A lack of privacy-preserving collaborative learning further limits decentralized adaptation across intersections. Table 1 shows that the research problems addressed in this study are the lack of a scalable, real-time, and intelligent traffic management framework that can combine predictive modeling, adaptive optimization, and secure distributed learning.

**Table 1:** Limitations of traditional traffic management systems and corresponding research needs addressed by GEC-DTSP.

Current Limitation	Advancement in GEC-DTSP
Centralized systems cause latency.	Edge–cloud distributed processing for real-time control
Poor spatiotemporal modeling	GCN + LSTM for short-term dynamics and Transformer-based forecasting for long-term traffic prediction
Absence of predictive intelligence	Integration of predictive modeling within the Digital Twin Engine for proactive congestion management
Delayed responses under growing demand	Continuous synchronization of real-time edge data with historical cloud data for faster decision support
Low scalability under data load	Hybrid Digital Twin architecture integrating edge–cloud for scalable, sustainable traffic infrastructure

### 1.3 Research strategy

Research architecture of GEC-DTSP integrates edge–cloud computing, predictive learning, and digital twin technology to facilitate intelligent traffic management. IoT-based roadside sensors yield streams of data that are processed at the edge devices with minimal latency for traffic analysis. GCN identifies spatial dependencies among road networks, and LSTM models learn temporal patterns of traffic flow. A Transformer predictor is employed to enhance long-term traffic prediction and facilitate strategic planning. A Digital Twin Engine at the cloud layer updates the real-time edge data by correlating it with historical data, keeping the virtual model of the traffic system current for monitoring and simulation. Building upon these predictive layers, an RL module dynamically optimizes traffic signal control, adaptive routing, and congestion mitigation by interacting with both physical and virtual environments. Experimental testing implements simulations and actual datasets, contrasting the enhancements in vehicle mean waiting time, congestion prediction accuracy, scalability, and resilience under missing-data conditions, and concludes that GEC-DTSP is an intelligent and sustainable traffic system.

### 1.4 Contributions

The main contributions of the study are:

- An edge–cloud integrated digital twin framework enabling coordinated traffic data processing and real-time system synchronization.
- A hybrid spatiotemporal learning model combining Graph Convolutional Networks (GCN), LSTM networks, and Transformer-based forecasting for multi-scale traffic prediction.
- A reinforcement learning-based control module for adaptive traffic signal optimization based on dynamic traffic states.
- A unified architecture for integrating spatial, temporal, and control-level intelligence in urban traffic systems.

The proposed framework centers on three validated components: (i) spatiotemporal traffic forecasting, (ii) adaptive traffic signal control utilizing reinforcement learning, and (iii) digital twin-based synchronization for real-time traffic state alignment. Conceptual advancements, including privacy-preserving collaborative learning and adaptive routing, are incorporated into the system design but are not practically implemented in the present work.

### 1.5 Research questions

RQ1: In what manner might spatiotemporal traffic relationships be proficiently characterized utilizing a hybrid GCN–LSTM–Transformer architecture?

RQ2: In what manner does the synchronization of edge-cloud digital twins enhance the accuracy of real-time traffic state estimate and prediction?

RQ3: To what degree can reinforcement learning improve adaptive traffic signal regulation in fluctuating congestion scenarios?

RQ4: What is the robustness of the suggested system in the presence of noise, incompleteness, and large-scale data?

### 1.6 Paper organization

The rest of the paper is followed by Section 2 which reviews the recent work on traffic prediction, and digital twin frameworks. Section 3 details the datasets and the proposed methodology. Section 4 gives experimental setup and evaluation metrics. Section 5 gives the discussion followed by Section 6 giving the limitation of the study. Finally, the conclusion is drawn in Section 7.

## 2. Related works

### 2.1 Conventional traffic prediction models

Sattarzadeh et al. (2025) [17] introduced a hybrid traffic flow forecasting model that combined ARIMA, Conv-LSTM, and a shuffle attention layer. The approach integrated spatiotemporal time-series analysis, utilizing statistics and deep learning, to address the nonlinear dynamics of traffic flow. The research demonstrated improved accuracy and resilience compared to single ARIMA or Conv-LSTM models. The drawback highlighted was limitations in scaling to a few smart city infrastructures without efficient model compression or edge deployment. Su et al. (2024) [18] had suggested a low-cost hybrid attention network for traffic forecasting in 5G transport systems. The approach used deep learning in combination with attention layers to capture temporal dynamics at lower computational expense. The results indicated higher accuracy and cost savings at moderate communication capacity, supporting the model’s application in real-time in 5G systems. The model had not been fully tested in heterogeneous or non-5G environments, which are still dominant in urban areas and pose challenges to widespread adoption.

Taher et al. (2025) [19] introduced a traffic congestion prediction technique based on filters that incorporated traffic signal movement in dynamic state estimation. The technique incorporated new filtering algorithms to combine real-time flow and real-time signal timing data, thereby improving estimation accuracy. Results validated enhanced responsiveness and improved congestion prediction compared to the existing practice. But it had the limitation that it was derived from highly accurate and pervasive traffic signal data. Jiang et al. (2025) [20] introduced an adaptive prediction model that can handle sparsely distributed traffic information on city networks. Adaptive learning processes were employed in the methodology to efficiently process sparse and random measurements. Greater precision than traditional static models was realized under incomplete or randomly sampled data. Precision, however, declined under heavy traffic fluctuations, where the adaptive updates were unable to detect sudden interruptions and demand changes. Sengupta et al. (2024) [21] developed a Bayesian model to quantify uncertainty and improve the overallizability of traffic forecasting models. The method integrated Bayesian inference with deep learning, providing prediction intervals alongside point forecasts. The presented results demonstrated greater robustness, reliability, and interpretability in the presence of noisy or uncertain data. However, the drawbacks included high computational cost and reduced inference speed, making it less suitable for real-time applications.

### 2.2 Edge and cloud-based traffic solutions

Reza et al. (2025) [22] proposed a city-level traffic signal control system based on TD-learning for autonomous vehicle deployment. The approach combined reinforcement learning and SUMO simulations to adjust traffic signal phases dynamically. Outcomes demonstrated a significant reduction in vehicle waiting time and improved traffic flow for simulated cases. Its limitation was its reliance solely on simulations, which lacked real-world deployment information, thereby reducing its near-term relevance to smart urban infrastructure. Medvei et al. (2025) [23] proposed DeepSIGNAL-ITS, an adaptive traffic signal control system for smart transportation based on deep learning. It utilized neural networks to process multi-source traffic signals and adaptively adjust signals. Performance led to the elimination of bottlenecks and enhanced vehicle throughput in simulation tests. Its limitation was that it required high-quality, synchronized, and multimodal input data, which are not typically found in most realistic real-world systems; hence, it lacked robustness against erroneous sensing for practical real-world deployment. Kan et al. (2024) [24] suggested optimizing urban traffic management by integrating YOLOv5 for real-time vehicle detection and DeepSORT for tracking within a digital twin framework. The framework analyzed real-time traffic video streams to ensure proper vehicle movement tracking, facilitating dynamic traffic analysis and sophisticated congestion management.

Liu et al. (2021) [25] proposed a smart traffic monitoring system based on computer vision and edge computing, enabling the real-time processing of video streams. It supported traffic density, congestion, and anomaly detection at edge devices with lower latency and lower network load. The model enhanced traffic observation responsiveness in urban areas, enabling timely interventions. However, its performance was limited by camera location, time-varying lighting, and occlusions. Accuracy decreased when traffic was dynamically or highly complex, limiting its potential for large-scale deployment across varied urban settings. Alkarim et al. (2024) [26] suggested ensemble learning-based algorithms for short-term traffic flow prediction for intelligent traffic systems. By integrating several prediction models, the method achieved greater predictive precision and robustness against failures in individual models. It presented enhanced traffic intelligence for real-time planning and management. The approach, however, increased computational complexity, necessitating greater computational capacity.

### 2.3 Digital Twin applications in urban traffic

Khadka et al. (2025) [27] developed Automated Traffic Signal Performance Measures (ATSPMS) in a digital twin simulation platform. The method offered virtual testing and optimization of traffic signal plans, enabling data-driven improvements in intersection efficiency. Through traffic simulation, it provided insights into reducing congestion and informed operational planning. Its weakness was a reliance on affluent, high-fidelity input data and iterative road network models, which are not always readily accessible. Llagostera-Brugarola et al. (2025) [28] proposed a Digital Twin framework for smart transportation in intercity settings, enabling predictive traffic simulation, incident response, and operational optimization. The framework combined sensor information with simulation models to improve traffic observation and planning. Though effective in intercity corridors, it was marred by scalability issues when extended to multi-complex urban networks with interdependent traffic relationships. These characteristics limited its application in gigantic city deployments, where data heterogeneity and changing conditions are the norm.

Li et al. (2024) [29] developed a driver risk-conscious smart mobility analytics platform using a digital twin to manage traffic in cities. It predicted hazardous driver behavior and optimized traffic flow to make roads safer and congestion a bare minimum. The system used real-time sensor data and driver-specific data to make decisions. Its use of personal driver data, however, raised privacy issues and limited its broader application, and its potential within heterogeneous real-world urban settings is limited. Fu et al. (2024) [30] proposed a digital twin platform for pedestrian safety warnings at an individual city intersection. The platform combined real-time sensor data with predictive modeling to warn pedestrians and drivers of potential collisions, enhancing safety. Simulation outputs verified it to be effective in minimizing hazards of accidents. Its dependence on quality targets and live data reduces resilience when sensors fail or provide inadequate data, limiting its practical use to larger metropolitan traffic networks.

Yanzhan Chen et al. [31] suggested the 3D Gaussian splatting (3D-GS) for intelligent 3D traffic accident reconstruction. To segment large-scale 3D point clouds, a clustering parameter stochastic optimization model and mixed-integer programming Bayesian optimization (MIPBO) method are suggested. 3D-GS suffers with visual rendering at night and in rain. In numerical trials, 3D-GS renders high-quality, seamless, real-time traffic accident scenarios with a structural similarity index of 0.90 across municipalities. Additionally, the MIPDBO method quickly converges, identifying optimal parameters in 3–5 iterations and achieving a high value of R2 0.8 on a benchmark cluster issue. Finally, the Gaussian Mixture Model with MIPBO distinguishes accident scene traffic components better than standard clustering techniques.

Yanzhan Chen et al. [32] proposed the Roadside LiDAR placement for cooperative traffic detection by a novel chance constrained stochastic simulation optimization approach. For Roadside LiDAR (RSL) placement, this study proposes a chance-constrained stochastic simulation-based optimization (SO) model to maximize mean Average Precision (mAP) with a budgeted number of RSLs and a chance constraint of ensuring a specific recall value under traffic uncertainties. Significantly, a data-driven deep learning strategy based on a high-fidelity co-simulator is used to evaluate an RSL placement plan, which is black-box, computationally expensive, and stochastic. These issues are addressed by a unique Gaussian Process Regression-based Approximate Knowledge Gradient (GPR-AKG) sampling approach. An RSL placement plan optimized by GPR-AKG achieves an outstanding mAP of 0.829 while meeting the chance limitation in numerical trials on a bi-directional eight-lane roadway, outperforming empirically developed alternatives. Under the optimal design, cooperative vehicle detection and tracking may reduce false alarms and missed detections from significant vehicle occlusions and create comprehensive and smooth vehicle trajectories. Analysis of detection coverage and average effective work time supports selecting center-mounted RSLs in the optimum plan. Deploying 20 RSLs in the optimum design is scientifically justified based on mAP balancing research.

Jingke Yan et al. [33] presented the a multimodal arc detection network based on denoising diffusion probabilistic models (DDPMs-MILNet) for arc detection in railway systems with limited data. To learn complex image characteristics, a DDPM is pretrained on many unlabeled photos. This model extracts features and fine-tunes a hierarchical variation semantic decoder to improve performance under small-sample settings and reduce dependency on labeled datasets. Based on this, an audiovisual semantic decoder uses audio signals as semantic cues to provide visual characteristics with modality information. This method decreases the model’s dependence on visual input and allows it to detect the arc’s visual target even when the item is not seen and heard, relieving the problems of small sample numbers. DDPM-MILNet performs well with little data in complicated railway settings, suggesting it might be used for railway system condition monitoring and anomaly identification.

Xin Wang et al. [34] introduced the adaptive fused domain-cycling variational generative adversarial network (AFDVGAN) for machine fault diagnosis under data scarcity. First, a smooth-regularized variational framework stabilizes latent space representation, increasing synthetic data structural consistency and training stability. Second, a ratio-controlled domain-cycling mechanism dynamically coordinates feature transfer between spatial, time-frequency, and frequency domains to improve multi-domain feature modeling and data synthesis. Finally, a multi-metric guided adaptive data fusion technique fuses synthetic and real data using statistical and time-frequency metrics to improve diagnostic model decision-making accuracy. AFDVGAN produces better synthetic data than normal and state-of-the-art approaches for electric locomotive and high-speed aerospace bearing case studies. Data fusion improves locomotive diagnostic accuracy to 99.81% and aerospace bearing accuracy to 99.16%.

Hui Wang et al. [35] discussed the Generalized Koopman Neural Operator for Data-Driven Modeling of Electric Railway Pantograph–Catenary Systems. The author presented a new generalized Koopman neural operator (GKNO) implemented using an autoencoder and an upgraded Transformer to describe complicated nonlinear dynamic systems with significant degrees of freedom. It has observable, evolution, and invertible functions. As an embedding model, the encoder transfers the original system’s state variables into observable space with linear dynamics. Using an autoregressive task, an enhanced Transformer model learns the embedding of space evolution function. Finally, using the embedding space, the decoder reconstructs the original system’s state variables.

HuanZhong Sun et al. [36] deliberated the Spatio-Temporal Graph Neural Network for Traffic Prediction Based on Adaptive Neighborhood Selection (STGNN-ANS). STGNN-ANS filters undesirable neighbors to create a new graph structure for more flexibility. A spatio-temporal serial module of STGNN-ANS uses bidirectional learning of bidirectional long short-term memory (BiLSTM) and the graph convolution network (GCN) enhanced by self-attention mechanism to capture traffic data’s spatio-temporal dependence and achieve superior prediction accuracy in both short- and long-range scenarios.

Qinyao Luo et al. [37] developed the Long-Short Term Transformer-based spatiotemporal neural network for traffic flow forecasting (LSTTN). The model learns compressed and contextual subseries temporal representations from long historical series by pretraining a masked subseries Transformer to infer the content of masked subseries from a small portion of unmasked subseries and their temporal context. After learning representations, stacked 1D dilated convolution layers extract long-term trend and dynamic graph convolution layers extract periodic features. A short-term trend extractor helps LSTTN learn fine-grained short-term temporal cues for time-step level prediction. Final predictions are made by LSTTN combining long-term trend, periodic characteristics, and short-term features. Experimental results on four real-world datasets reveal that the LSTTN model improves 60-minute-ahead long-term forecasting by 5.63% to 16.78% over baseline models.

Saira Karim et al. [38] investigated the Dynamic Spatial Correlation in Graph WaveNet for Road Traffic Prediction. This work uses attention mechanisms to calculate time-domain attention scores for the self-adaptive adjacency matrix to add dynamic spatial dependencies into the Graph WaveNet model. We compared the computation cost of our graph attention network model with multi-head attention to Graph WaveNet for up to 60 minutes. The best 60-min forecast model was ours, with root-mean-square error decreasing 3.4% and 4.76% on PEMS-BAY and METR-LA datasets, respectively. Attention score calculation increases model training time. Table 2 summarizes the related work.

**Table 2:** Summary of the related works.

Ref	Method Type	Predictive Capability	Digital Twin	Evaluation	Dataset / Data Source
[17]	Hybrid DL	Short-term traffic flow	☒	Simulation	Real-world urban traffic + synthetic data
[18]	Deep Learning	Short-term/5G traffic	☒	Real-time	5G traffic dataset / Signal logs
[19]	Filter-based	Congestion prediction	☒	Dynamic Estimation	Traffic signal & loop detector data
[20]	Adaptive Model	Traffic prediction	☒	Simulation	Urban road network traffic datasets
[21]	Bayesian	Uncertainty quantification	☒	Simulation	Public urban traffic datasets
[22]	TD-Learning	☒	☒	SUMO Simulation	SUMO simulated autonomous vehicle traffic
[23]	DeepSIGNAL	☒	☒	Simulation	Urban traffic datasets (multi-source)
[24]	CV + Tracking	Vehicle monitoring	☑	Real-time	Urban traffic video datasets
[25]	CV + Edge	Traffic monitoring	☒	Real-time	CCTV / traffic camera streams
[26]	Ensemble Learning	Traffic flow prediction	☒	Simulation	Public urban traffic datasets
[27]	Digital Twin	☒	☑	Simulation	Traffic simulation & sensor datasets
[28]	Digital Twin	Predictive modeling	☑	Simulation	Interurban traffic datasets
[29]	Digital Twin	Risk-aware analytics	☑	Real-time	Vehicle & driver behavior datasets
[30]	Digital Twin	☒	☑	Real-time	Pedestrian & intersection traffic datasets

### 2.4 Research gap

Despite progress in intelligent transport systems, current traffic management methods remain limited by static signal timing, centralized processing, and inadequate predictive modeling. Current studies using hybrid deep models, such as ARIMA–ConvLSTM [17] and attention-based neural networks [18], achieve better short-term predictions but lack the ability to model nonlinear spatiotemporal dependencies, which are critical for real-time adaptation. Reinforcement-based and signal intelligence architectures [22,23] provide localized control without global coordination or scalability. Digital twin-based deployments [27–29] offer virtual monitoring but are hindered by synchronization delays and reliance on high-latency cloud infrastructure. Therefore, the most significant research gap is the development of a GEC-DTSP that integrates edge intelligence, spatiotemporal learning (GCN–LSTM–Transformer), and real-time digital twin synchronization to achieve adaptive, scalable, and sustainable traffic management.

## 3. Methods and methodology

**Fig 1:**
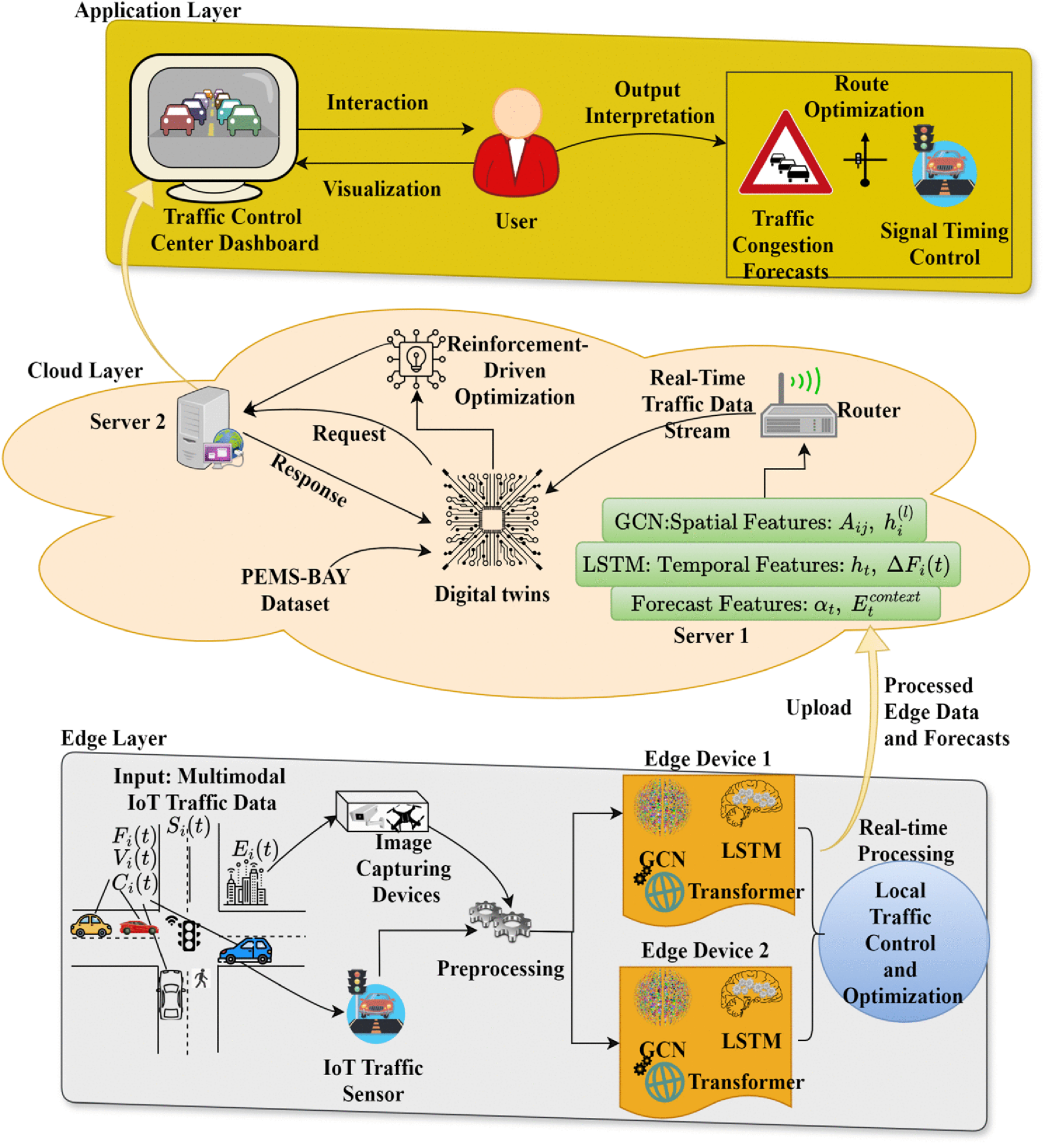
System architecture of the GEC-DTSP methodology.

The GEC-DTSP aims to enhance real-time traffic control in cities by leveraging combined edge computing, deep learning, and digital twin technologies. The system architecture in Fig 1 leverages low-latency data processing via edge devices and mass-scale simulation and coordination via cloud servers. IoT traffic sensors provide an input data stream that is continuously processed at the edge layer using GCN to represent spatial patterns involving interacting road networks. Temporal trends and traffic movement are modeled using LSTM models, while long-term prediction is facilitated using Transformer-based predictors for studying congestion patterns. The cloud-level Digital Twin Engine enables coordinated virtual simulation of the physical traffic network by combining real-time edge data with historical data for predictive analysis, scenario simulation, and strategic decision-making. This multi-modal system collectively brings responsiveness, scalability, and sustainability to intelligent transportation infrastructure.

### 3.1 Dataset explanation

The Kaggle Smart Traffic Management Dataset [39] is a small yet useful dataset comprising approximately 2,000 rows and 12 features that describe city traffic conditions. It includes variables such as vehicle count, signal timing, lane occupation, and other contextual data, making it ideal for prototyping traffic analysis methods at a small enough scale. It is beneficial for investigating algorithmic feasibility, prototyping, and rapid experimentation in environments with constrained computational resources. Conversely, the PEMS-BAY dataset [40], available on Zenodo, is a real-world benchmark of high-volume data collected from loop detectors in the San Francisco Bay Area freeway network. It comprises high-resolution speed and volume observations from hundreds of sensors, along with an adjacency graph representing the road network’s spatial topology.

**Fig 2:**
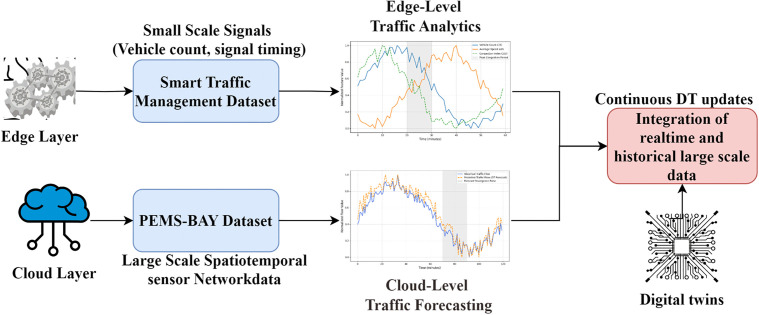
Layered architecture for Edge-Cloud Digital Twin Traffic Management using Kaggle Smart Traffic Dataset and PEMS-BAY Dataset.

The proposed framework uses a dual-dataset approach to independently support the localized traffic signal control and the large-scale modeling of traffic dynamics. It uses the Kaggle Smart Traffic Management dataset with about 2,000 samples including 12 traffic-related characteristics as a basis to control traffic signal lights at the intersection level. It entails fine-grained data, including the number of vehicles, the presence of a lane, the length of a queue, and even the state of a signal phase, which are directly applicable to an adaptive signal optimization approach to urban intersections. PEMS-BAY on the other hand is integrated to model the large-scale propagation of macroscopic traffic flow and long-range spatiotemporal dependencies across large-scale road networks. This data is sensor data of 325 loop detectors installed throughout the San Francisco Bay Area freeway network, with 5-minute sampling interval. Though originally intended as a highway traffic forecasting tool, it is not directly applied in training intersection level signal control. Rather, it helps to learn the patterns of evolution of global traffic, spatial dependencies of distributed sensors, and temporal continuity of traffic flow, which are crucial in the calibration of the predictive modeling layer of the digital twin. Fig 2 shows tiered integration of data sets for intelligent traffic management.

In the Edge Layer, the Kaggle Smart Traffic Management Data Set delivers small-scale, real-time attributes of vehicle volumes, signal timing, and intersection queues for local processing. The Cloud Layer leverages large-scale PEMS-BAY data, including loop detector speeds, traffic volume flow, and sensor network occupancy rates, across a broad sensor network. Lastly, the Digital Twin Layer synchronizes edge attributes with historical cloud-based data in real time to support adaptive traffic signal control, congestion forecasting, and dynamic urban mobility planning.

### 3.2 Data acquisition

The Data Acquisition module serves as the foundational layer that gathers multimodal traffic data from IoT-enabled infrastructure deployed across intersections. The sensing network consists of vehicular detectors, camera-based vision sensors, and signal controllers, all integrated through an edge gateway. These devices continuously monitor critical traffic parameters, including vehicle count, speed, flow rate, and signal phase, serving as the primary inputs for real-time modeling and control. The raw data stream from each sensor node i at time t can be represented as a multivariate feature vector, as shown in equation 1.


$$\begin{array}{c} {\text{X}}_{\text{i}}(\text{t})=\;\begin{array}{c} \left\{{\text{C}}_{\text{i}}(\text{t}),{\text{V}}_{\text{i}}(\text{t}),{\text{F}}_{\text{i}}(\text{t}),{\text{S}}_{\text{i}}(\text{t}),{\text{E}}_{\text{i}}(\text{t})\right\}\\ {\text{C}}_{\text{i}}(\text{t})=\sum_{\text{j}=\text{1}}^{{\text{M}}_{\text{i}}}\delta_{\text{ij}}(\text{t})\\ {\text{V}}_{\text{i}}(\text{t})=\frac{\text{1}}{{\text{C}}_{\text{i}}(\text{t})}\sum_{\text{k}=\text{1}}^{{\text{C}}_{\text{i}}(\text{t})}{\text{v}}_{\text{ik}}(\text{t})\\ {\text{F}}_{\text{i}}(\text{t})=\frac{{\text{C}}_{\text{i}}(\text{t})}{\Delta \text{t}}\\ {\text{S}}_{\text{i}}(\text{t})=\left\{ \begin{array}{c} \text{1},\;\text{if the signal is GREEN}\\ \text{0},\;\text{if the signal is RED or YELLOW} \end{array}\right.\;\\ {\text{E}}_{\text{i}}(\text{t})=\alpha_{\text{1}}{\text{T}}_{\text{i}}(\text{t})+\alpha_{\text{2}}{\text{H}}_{\text{i}}(\text{t})+\alpha_{\text{3}}{\text{R}}_{\text{i}}(\text{t}) \end{array}\} \end{array}$$ (1)

where ${\text{C}}_{\text{i}}(\text{t})$ refers to total number of vehicles detected at the intersection i at time t. ${\text{V}}_{\text{i}}(\text{t})$ is the average vehicle speed, ${\text{F}}_{\text{i}}(\text{t})$ is the traffic flow rate (vehicles per minute), ${\text{S}}_{\text{i}}(\text{t})$ denotes the current signal phase, and ${\text{E}}_{\text{i}}(\text{t})$ corresponds to environmental factors such as weather or illumination at the time t. ${\text{M}}_{\text{i}}$ is the total detection zones or camera frames at the intersection i. $\begin{array}{c} \delta_{\text{ij}}(\text{t})=\left\{ \begin{array}{c} @l\text{1},\;\text{if a vehicle is detected in zone }\text{j}\text{ at time }\text{t}\\ \text{0},\;\text{otherwise} \end{array}\;\;\;,\right.\; \end{array}$ ${\text{v}}_{\text{ik}}(\text{t})$ refers to the instantaneous velocity of the ${\text{k}}^{\text{th}}$ vehicle at the intersection i at time t. ${\text{F}}_{\text{i}}(\text{t})$ is the flow rate (vehicles per unit time). Δt denoted time window or observation interval (e.g., 30 s or 1 min). ${\text{T}}_{\text{i}}(\text{t})$ is the temperature at the location i, ${\text{H}}_{\text{i}}(\text{t})$ is the humidity, ${\text{R}}_{\text{i}}(\text{t})$ is the rainfall intensity, $\alpha_{\text{1}},\alpha_{\text{2}},\alpha_{\text{3}}$ refers to the normalization coefficients or learned weights. The sampling frequency defines the data acquisition rate ${\text{f}}_{\text{s}}$, ensuring temporal resolution consistency, ${\text{T}}_{\text{s}}=\frac{\text{1}}{{\text{f}}_{\text{s}}}$, where ${\text{T}}_{\text{s}}$ denotes the sampling interval between consecutive measurements. To maintain synchronization across heterogeneous sensors, a timestamp alignment mechanism is applied, ensuring that, ${\text{t}}_{\text{align}}=\text{min}(|{\text{t}}_{\text{cam}}-{\text{t}}_{\text{loop}}|,|{\text{t}}_{\text{ctrl}}-{\text{t}}_{\text{env}}|)$. This synchronization ensures spatiotemporal consistency across different data sources before they are forwarded to the edge layer for preprocessing. To ensure robust temporal synchronization across heterogeneous traffic sensors, a tolerance-window-based alignment strategy is adopted. Instead of selecting the minimum timestamp difference directly, a bounded temporal matching function is used: $t_{\text{align}}=\text{arg}\underset{t_{j}\in T}{\text{min}}\left(|t_{i}-t_{j}|\right),\text{subject to }|t_{i}-t_{j}|\leq \Delta T$*,* where ΔT represents the maximum allowable synchronization window (e.g., 5–10 seconds depending on sensor type). If no match exists within this window, linear interpolation is applied between adjacent timestamps to preserve continuity in the data stream.

### 3.3 Edge processing

Each edge node i receives a multidimensional traffic feature vector defined as equation 1. To align heterogeneous sensor readings with standard data ranges from the Kaggle Smart Traffic Management Dataset, a normalization (${\tilde{\text{X}}}_{\text{i},\text{k}}(\text{t})$) process is performed. To improve data credibility and eliminate the temporal noise effect, a smoothing filter is employed using a moving average function (${\hat{\text{X}}}_{\text{i},\text{k}}(\text{t})$). Based on the processed signals, a localized congestion index is computed to capture the dynamic traffic density ($\Gamma_{\text{i}}(\text{t})$). To facilitate efficient communication between the edge and cloud layers, the processed data undergoes feature compression using a lightweight encoder ${\text{Z}}_{\text{i}}(\text{t})$ as in equation 2.


$$\begin{array}{c} {\text{Z}}_{\text{i}}(\text{t})=\left\{ \begin{array}{c} @c{\text{f}}_{\text{enc}}({\hat{\text{X}}}_{\text{i}}(\text{t}),\Gamma_{\text{i}}(\text{t}))\\ {\hat{\text{X}}}_{\text{i},\text{k}}(\text{t})=\frac{\text{1}}{\text{W}}\sum_{\tau =\text{t}-\text{W}+\text{1}}^{\text{t}}{\tilde{\text{X}}}_{\text{i},\text{k}}(\tau )\\ {\tilde{\text{X}}}_{\text{i},\text{k}}(\text{t})=\frac{{\text{X}}_{\text{i},\text{k}}(\text{t})-\overset{\text{min}}{\underset{\text{k}}{\text{X}}}}{\overset{\text{max}}{\underset{\text{k}}{\text{X}}}-\overset{\text{min}}{\underset{\text{k}}{\text{X}}}}\\ \Gamma_{\text{i}}(\text{t})=\alpha_{\text{1}}\frac{{\text{C}}_{\text{i}}(\text{t})}{{\text{C}}_{\text{max}}}+\alpha_{\text{2}}\left(\text{1}-\frac{{\text{V}}_{\text{i}}(\text{t})}{{\text{V}}_{\text{max}}}\right)+\alpha_{\text{3}}\frac{{\text{F}}_{\text{i}}(\text{t})}{{\text{F}}_{\text{max}}}\\ {\text{C}}_{\text{max}}\in [\text{150},\text{250}]\text{veh}/\text{km}/\text{lane}\\ {\text{V}}_{\text{max}}\in [\text{60},\text{120}]\text{km}/\text{h}\\ {\text{F}}_{\text{max}}\in [\text{1800},\text{2400}]\text{veh}/\text{hr}/\text{lane} \end{array}\right.\; \end{array}$$ (2)

where ${\text{X}}_{\text{i},\text{k}}(\text{t})$ is the raw feature value, $\overset{\text{min}}{\underset{\text{k}}{\text{X}}}\text{ and }\overset{\text{max}}{\underset{\text{k}}{\text{X}}}$ are feature-wise minimum and maximum dataset-calculated values, and ${\tilde{\text{X}}}_{\text{i},\text{k}}(\text{t})$ is the normalised value that provides equal scaling across various intersections. where W represents the smoothing window size and $\;{\hat{\text{X}}}_{\text{i},\text{k}}(\text{t})\;$ is the smoothed observation. $\Gamma_{\text{i}}(\text{t})$ represents the congestion degree at the node i, $\alpha_{\text{1}},\alpha_{\text{2}},\alpha_{\text{3}}$ are weight coefficients indicating the contribution of vehicle count, speed, and flow rate, respectively, while ${\text{C}}_{\text{max}},{\text{V}}_{\text{max}},$ and ${\text{F}}_{\text{max}}$ denote their respective maximum observed values. A higher $\Gamma_{\text{i}}(\text{t})$ value implies increased congestion severity. ${\text{f}}_{\text{enc}}(\cdot )$ represents the encoding function, often implemented through a compact CNN or autoencoder, and ${\text{Z}}_{\text{i}}(\text{t})$ is the latent representation transmitted to the cloud-based Digital Twin Engine. The spatiotemporal feature encapsulation reduces bandwidth usage significantly without compromising the critical spatiotemporal aspects of traffic.

**Fig 3:**
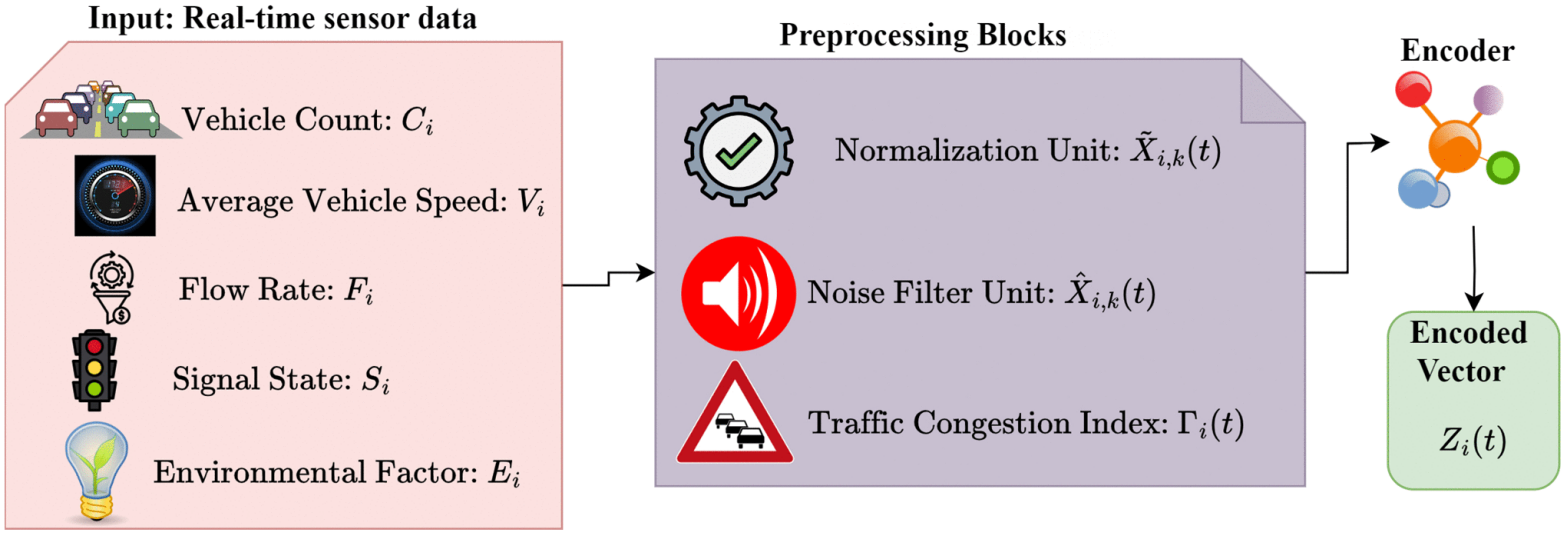
Edge node data preprocessing in GEC-DTSP.

Fig 3 shows the edge node data preprocessing in GEC-DTSP. The edge processing phase, therefore, undertakes an integrated process of real-time data normalization, temporal-domain data smoothing, congestion estimation, and feature encoding—thereby improving computational efficiency and sending only high-value, structured data to the cloud layer for synchronization and predictive modeling. The pre-encoded edge features, preprocessed, constitute the fundamental input of the hybrid GCN–LSTM–Transformer model. The feature-compressed representations of these features retain important spatial and temporal details while minimizing redundancy for low-cost transmission over the cloud. GCN captures spatial interdependencies between intersections, the LSTM captures temporal traffic patterns, and the Transformer uses attention mechanisms for multi-horizon prediction.

### 3.4 Spatiotemporal modeling and predictive learning in GEC-DTSP

The hybrid deep model of the GEC-DTSP architecture combines GCNs, LSTM networks, and a Transformer-based predictor to capture spatial dependencies within the traffic network and temporal evolution. GCN captures spatial relationships between road junctions, LSTM captures sequential temporal changes, and the Transformer captures long-term dependencies through self-attention mechanisms.

#### 3.4.1 Graph convolutional network for spatial modeling.

**Fig 4:**
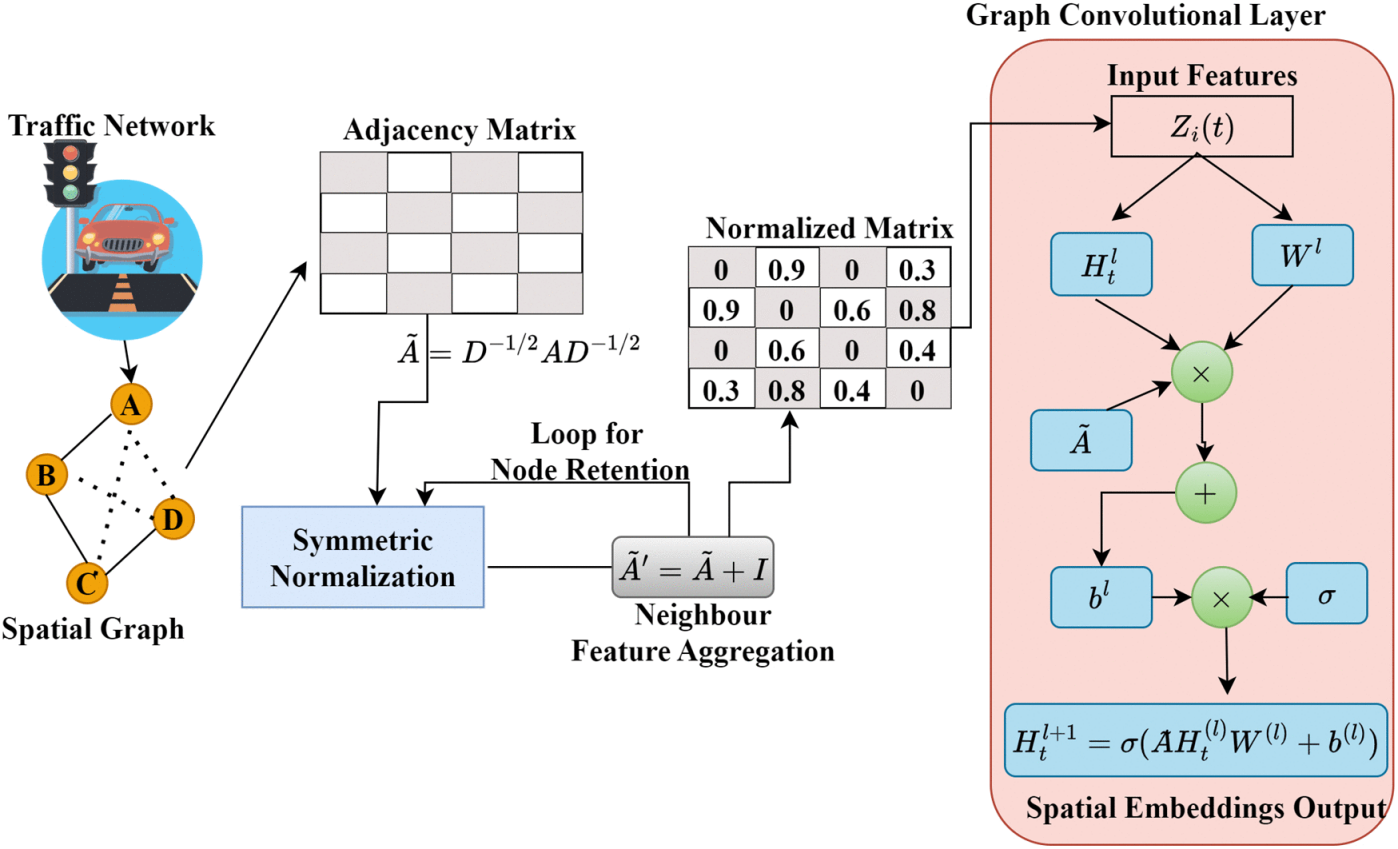
The GCN-based spatial modeling process in GEC-DTSP.

GCNs offer a principled approach to capturing the propagating patterns of traffic states across interconnected road segments, treating the road network as a graph. A rigorous mathematical definition of GCNs for representing spatial traffic dependencies among cities is provided in this section. GCN learns spatial correlations between neighboring road junctions by structuring the traffic network as a graph, where G=(V,E,A) wherein V are the nodes (junctions), E are the roads connecting them, and A is the adjacency matrix representing the spatial relationships. Each node feature vector ${\text{X}}_{\text{t}}\in R^{\text{N}\times \text{F}}$ is made up of multivariate traffic conditions, including vehicle volume, speed, and flow rate at time t. A weighted adjacency matrix is constructed based on spatial proximity and connections. Formally, this can be written as in equation 3.


$$\begin{array}{c} {\text{A}}_{\text{ij}}=\left\{ \begin{array}{c} @c{\text{w}}_{\text{ij}}\cdot {\text{1}}_{\text{i},\text{j}}\in \text{E}\\ \text{where }{\text{1}}_{\text{i},\text{j}}\in \text{E}=\left\{ \begin{array}{c} @l\text{1},\text{ if }(\text{i},\text{j})\in \text{E }\\ \text{0},\text{otherwise} \end{array}\right.\;\;\;\;\;\;\;\;\;\;\;\;\;\;\;\;\\ {\text{w}}_{\text{ij}}=\text{exp}(-\frac{\overset{\text{2}}{\underset{\text{ij}}{\text{d}}}}{\text{2}\sigma^{\text{2}}}) \end{array}\right.\; \end{array}$$ (3)

where ${\text{w}}_{\text{ij}}$ represents the connection weight between nodes i and j. is the Euclidean distance between road segments (or intersections) i and j, and σ is a bandwidth parameter controlling the spatial decay of influences. Typically, ${\text{w}}_{\text{ij}}$ is set to zero for distances exceeding a threshold distance τ to capture only local spatial interactions. To ensure numerical stability during graph convolution operations, the adjacency matrix is normalized using the symmetric normalization scheme as in equation 4.


$$\begin{array}{c} \tilde{\text{A}}=\left\{ \begin{array}{c} @c{\text{D}}^{-\text{1}/\text{2}}\text{A}{\text{D}}^{-\text{1}/\text{2}}\\ \text{ D}\in R^{\text{n}\times \text{n}}\\ {\text{D}}_{\text{ii}}=\sum_{\text{j}=\text{1}}^{\text{n}}{\text{A}}_{\text{ij}},\;{\text{D}}_{\text{ij}}=\text{0for i}\neq \text{j}\\ {\tilde{\text{A}}}^{\prime }=\tilde{\text{A}}+\text{I} \end{array}\right.\; \end{array}$$ (4)

where D is the degree matrix, I is the identity matrix. Normalization ensures that features from neighboring nodes are properly scaled by their node degrees, preventing extreme value amplification during message passing. In practice, the normalized adjacency matrix (${\tilde{\text{A}}}^{\prime }$) is often augmented with self-loops. Self-loops enable each node to retain its own information during graph convolution, facilitating better feature learning. The graph convolution operation for the ${\text{l}}^{\text{th}}$ layer is expressed as in equation 5.


$$\begin{array}{c} \overset{\left(\text{l}+\text{1}\right)}{\underset{\text{t}}{\text{H}}}=\left\{ \begin{array}{c} @c\sigma (\tilde{\text{A}}\overset{\left(\text{l}\right)}{\underset{\text{t}}{\text{H}}}{\text{W}}^{\left(\text{l}\right)}+{\text{b}}^{\left(\text{l}\right)})\\ \overset{\left(\text{l}\right)}{\underset{\text{t}}{\text{H}}}\in R^{\text{N}\times {\text{F}}_{\text{l}}}\\ {\text{W}}^{\left(\text{l}\right)}\in R^{{\text{F}}_{\text{l}}\times {\text{F}}_{\text{l}+\text{1}}}\\ \tilde{\text{A}}={\text{D}}^{-\frac{\text{1}}{\text{2}}}(\text{A}+\text{I}){\text{D}}^{-\frac{\text{1}}{\text{2}}} \end{array}\right.\; \end{array}$$ (5)

where $\overset{\left(\text{l}\right)}{\underset{\text{t}}{\text{H}}}$ denotes node feature matrix at layer l, ${\text{W}}^{\left(\text{l}\right)}$ is the trainable weight matrix, ${\text{b}}^{\left(\text{l}\right)}$ refers to the bias vector, σ(·) symmetrically normalized adjacency matrix, D is the degree matrix. This operation aggregates spatial information from neighboring nodes, allowing each intersection to learn traffic dependencies from surrounding regions. The resulting $\overset{\left(\text{L}\right)}{\underset{\text{t}}{\text{H}}}$ encapsulates spatial embeddings used for subsequent temporal modeling. Fig 4 illustrates the GCN-based spatial modeling pipeline of GEC-DTSP. The road network is modeled as a weighted graph with intersections as nodes and roads as edges. The adjacency matrix is normalized and augmented with self-loops for stability. During graph convolution, every node gathers its neighbors’ spatial features to create spatial embeddings that capture inter-road dependencies for further temporal modeling.

#### 3.4.2 Long Short-Term Memory (LSTM) for temporal modeling.

As GCNs can accurately capture spatial relations at a single point in time, city traffic networks exhibit significant temporal dynamics that need to be modeled separately. Traffic volumes vary with characteristic patterns: morning rush-hour peaks, midday troughs, afternoon peak-hour spikes, and evening spinnings LSTM networks are specifically designed to learn long-term temporal correlations and avoid the vanishing gradient problem of traditional recurrent neural networks. The LSTM network retains temporal dependencies in traffic dynamics by iteratively processing space-encoded features from the GCN. It retains nonlinear relationships in traffic flow evolution, signal patterns, and congestion spreading via recurrent gating mechanisms, thereby preventing vanishing gradients. The forward computation of an LSTM unit is defined as in equation 6.


$$\begin{array}{c} \begin{array}{cc} {\text{i}}_{\text{t}} & =\sigma \left({\text{W}}_{\text{i}}{\text{H}}_{\text{t}}+{\text{U}}_{\text{i}}{\text{h}}_{\text{t}-\text{1}}+{\text{b}}_{\text{i}}\right),\text{ input gate}\\ {\text{f}}_{\text{t}} & =\sigma \left({\text{W}}_{\text{f}}{\text{H}}_{\text{t}}+{\text{U}}_{\text{f}}{\text{h}}_{\text{t}-\text{1}}+{\text{b}}_{\text{f}}\right),\text{ forget gate}\\ {\text{o}}_{\text{t}} & =\sigma \left({\text{W}}_{\text{o}}{\text{H}}_{\text{t}}+{\text{U}}_{\text{o}}{\text{h}}_{\text{t}-\text{1}}+{\text{b}}_{\text{o}}\right),\text{ output gate}\\ {\tilde{\text{c}}}_{\text{t}} & =\text{tanh}\left({\text{W}}_{\text{c}}{\text{H}}_{\text{t}}+{\text{U}}_{\text{c}}{\text{h}}_{\text{t}-\text{1}}+{\text{b}}_{\text{c}}\right),\;\\ {\text{c}}_{\text{t}} & ={\text{f}}_{\text{t}}⊙{\text{c}}_{\text{t}-\text{1}}+{\text{i}}_{\text{t}}⊙{\tilde{\text{c}}}_{\text{t}},\text{ cell state}\\ {\text{h}}_{\text{t}} & ={\text{o}}_{\text{t}}⊙\text{tanh}\left({\text{c}}_{\text{t}}\right),\text{ hidden state} \end{array} \end{array}$$ (6)

where ${\text{H}}_{\text{t}}$ refers to the GCN-derived input at time t, ${\text{h}}_{\text{t}}$ is the hidden state representing temporal feature encoding, ${\text{c}}_{\text{t}}$ is the cell state maintaining long-term memory, ${\text{i}}_{\text{t}},{\text{f}}_{\text{t}},{\text{o}}_{\text{t}}$ refers to the input, forget, and output gates controlling information flow, σ(·) is the sigmoid activation, ⊙ is the element-wise multiplication, ${\text{W}}_{*},{\text{U}}_{*},{\text{b}}_{*}$ are the trainable matrices and biases for each gate. The LSTM output sequence ${\text{h}}_{\text{t}}$ serves as input to the Transformer predictor, enabling long-term forecasting with attention-based weighting over multiple time horizons. The cell state update rule is critical to understanding LSTM’s effectiveness as in equation 7.


$$\begin{array}{c} {\text{C}}_{\text{t}}=\left\{ \begin{array}{c} @c{\text{f}}_{\text{t}}⊙{\text{C}}_{\text{t}-\text{1}}+{\text{i}}_{\text{t}}⊙{\tilde{\text{C}}}_{\text{t}}\\ {\text{f}}_{\text{t},\text{j}}\approx \left\{ \begin{array}{c} @c\text{1},\text{ if }{\text{j}}^{\text{th}}\text{ component of }{\text{C}}_{\text{t}-\text{1}}\text{is retained}\\ \text{0},\text{ if it is forgotten} \end{array}\right.\; \end{array}\right.\; \end{array}$$ (7)

The input gate ${\text{i}}_{\text{t}}$ also determines which elements of the candidate state ${\tilde{\text{C}}}_{\text{t}}$ are accumulated in. The additive update (plus sign) allows gradients to flow directly backward through the cell state without being attenuated by repeated multiplications. In traffic forecasting, gating mechanisms have intuitive interpretations as below.

- Forget Gate: Controls whether habits that were followed in the past are still good ones. When there is a major incident (e.g., a crash), the forget gate may be reset to zero to adopt new habits. When it is business-as-usual rush hour, it continues as before.
- Input Gate: Regulates the introduction of new observations. If new sensor input indicates a sudden change in traffic patterns, the input gate increases its strength in subsequent predictions.
- Output Gate: Specifies which portions of the saved cell state are relevant to the output for the present time step. There are different predictive features for traffic at night versus during rush hour; thus, the output gate selectively activates the relevant features.

**Fig 5:**
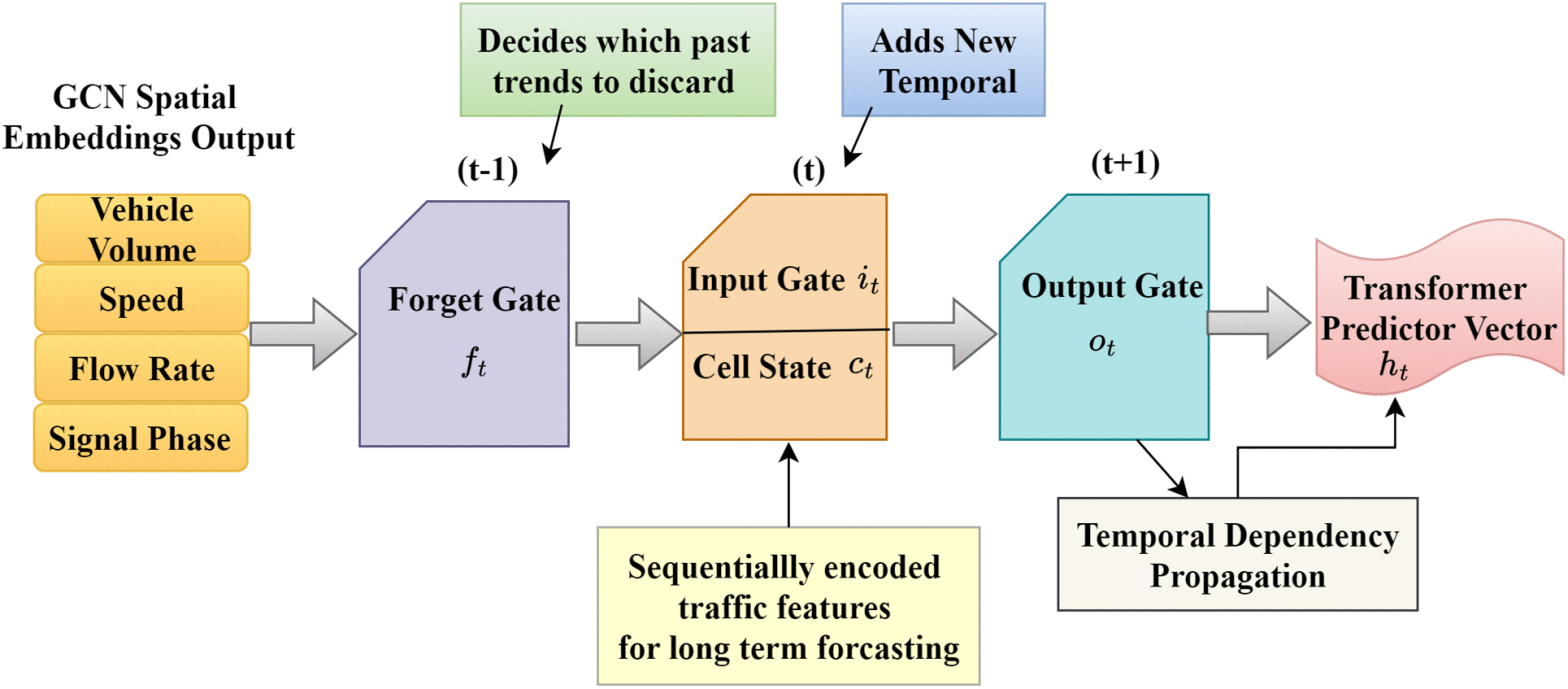
LSTM-based temporal modeling of traffic dynamics in GEC-DTSP.

By iterative training, the LSTM cell state acquires the ability to accept multi-dimensional temporal patterns such as periodic peak-hour timing patterns (peaking congestion time intervals identification), flow continuation patterns (acceleration or deceleration consistency assessment), anomaly detection cues (abnormal flow identification), and anticipation features (identification of forthcoming congestion with density gradients). These temporally encoded patterns enable resilient, adaptive prediction of short-term traffic behavior within the GEC-DTSP architecture shown in Fig 5.

GCN-learned spatial representations are fed into LSTM cells, with forget, input, and output gates controlling the flow of temporal information. Long-term traffic memory is remembered in cell state ${\text{C}}_{\text{t}}$, with varying congestion patterns disseminated across hidden states ${\text{h}}_{\text{t}}$. Gate activations adapt dynamically to the traffic phases shown in Table 3, allowing the model to predict rush-hour peaks, incident disruptions, and recovery responses.

**Table 3:** LSTM gate dynamics across different traffic phases in the GEC-DTSP.

Traffic Phase	Forget Gate (${𝐟}_{𝐭}$)	Input Gate (${𝐢}_{𝐭}$)	Output Gate (${𝐨}_{𝐭}$)	Cell State Value	Interpretation
Free Flow (Night)	Low (0.2–0.3)	Low (0.1–0.2)	Low (0.3–0.4)	Near zero	Minimal state retention; quiet observation mode
Pre-Rush (6–7 AM)	Medium (0.5–0.6)	High (0.7–0.8)	Medium (0.5–0.6)	Building up	Heavy input absorption; pattern anticipation begins
Peak Congestion (8 AM)	High (0.8–0.9)	High (0.7–0.8)	High (0.8–0.9)	Maximum	Full state retention; patterns strongly output
Incident (Sudden Drop)	Low (0.1–0.3)	Very High (0.9+)	High (0.8–0.9)	Shift	Rapid state reset; anomaly flag set
Recovery (After Incident)	Medium (0.4–0.6)	High (0.7–0.8)	High (0.7–0.8)	Declining	Gradual return to normal patterns
Off-Peak (Afternoon)	Low (0.3–0.4)	Low (0.2–0.3)	Medium (0.4–0.5)	Low	Limited memory; stable flow mode

Table 3 shows the dynamic behavior of the internal gates and state values of the LSTM cell across different stages of traffic. At free flow, all gate activations are low, holding very little information. During pre-rush, the input gate dominates as the model begins to detect early signs of congestion. Maximum congestion produces maximum activation across all gates, maintaining maximum memory retention and prediction output. The recovery phase demonstrates progressive state stabilization, and off-peak times return to low-gate operation, indicating normal traffic patterns.

**Fig 6:**
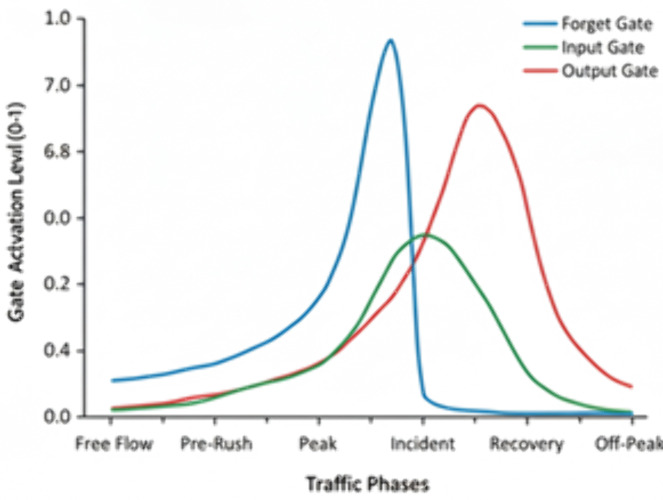
LSTM gate activation dynamics across traffic phases.

Fig 6 depicts the temporal dynamics of LSTM gate activation to various traffic phases in the GEC-DTSP model. The Forget Gate (blue) is highly active during incidents, reflecting the swift forgetting of old temporal patterns and adjustment to unexpected traffic interference. The Input Gate (green) increases its activity from pre-rush through to restoration, reflecting discrimination in the absorption of new congestion information as situations change. The Output Gate (red) is highest during the post-incident and recovery periods, capturing new temporal expectations for decongestion. Taken together, these dynamics reveal how the LSTM unit effectively controls memory persistence, information influx, and predictive output to capture the intricate temporal interdependencies of urban traffic flow.

#### 3.4.3 Transformer-based predictive forecasting for long-term traffic prediction.

To harmoniously represent spatial, temporal, and long-range dependencies, the LSTM network sequentially encodes its features and presents them to the Transformer predictor. The LSTM captures short-term sequential dependencies and produces temporally contextualized embeddings at each time step. These embeddings still maintain both spatial information (derived from GCN) and short-term dynamics (derived from LSTM). Pseudocode 1 provides the integration process of the hybrid GCN–LSTM–Transformer pipeline.

Pseudocode 1: Integration process of the hybrid GCN–LSTM–Transformer pipeline.

Input: ${\text{X}}_{\text{i}}(\text{t})$— traffic feature input for node i at time t, A— adjacency matrix of spatial graph connectivity

Output: ${\hat{\text{T}}}_{\text{i}}(\text{t}+\Delta \text{t})$— predicted traffic state at future time (t + Δt)

1: procedure Transformer_Predictive_Forecast (${\text{X}}_{\text{i}}(\text{t}),\text{A}$)

2:  # Step 1: Spatial Feature Extraction

3:   $Z_{i}(t)\leftarrow \text{GCN}(X_{i}(t),A;W_{gcn})$  // extracts spatial dependencies

4:  # Step 2: Short-Term Temporal Encoding

5:   $H_{t}\leftarrow \text{LSTM}(Z_{i}(t);W_{lstm})$// produces temporal embeddings [h_1_, h_2_, …, h_T]

6:  # Step 3: Transformer Attention for Long-Term Forecasting

7:   for each head h = 1 to H do

8:    $Q_{h}\leftarrow H_{t}W_{Q}[h];\;\;K_{h}\leftarrow H_{t}W_{K}[h];\;\;V_{h}\leftarrow H_{t}W_{V}[h]$

9:    $\;{\text{Attn}}_{h}\leftarrow \text{Softmax}\;\left(\frac{Q_{h}\overset{T}{\underset{h}{K}}}{\sqrt{d_{k}}}\right)V_{h}$

10:   end for

11:   $F_{multi}\leftarrow \text{Concat}\left({\text{Attn}}_{\text{1}},\ldots ,{\text{Attn}}_{H}\right)W_{o}$

12:   $C_{t}\leftarrow \text{LayerNorm}\left(H_{t}+F_{multi}\right)$

13:   $C_{t}\leftarrow \text{FeedForward}\left(C_{t}\right)$

14:   $C_{t}\leftarrow \text{LayerNorm}\left(C_{t}+\text{FeedForward}\left(C_{t}\right)\right)$

15:  # Step 4: Forecast Generation

16:   ${\hat{T}}_{i}(t+\Delta t)\leftarrow \text{Dense}(\overset{last}{\underset{t}{C}})$  // final future traffic prediction

17:   return ${\hat{T}}_{i}(t+\Delta t)$

18: End procedure

Let the input to the Transformer be a sequence of spatiotemporally encoded vectors $H_{t}=[h_{\text{1}},h_{\text{2}},\ldots ,h_{T}]$, where each $h_{t}\in R^{d}$ is the time t fused GCN–LSTM embedding. Scaled dot-product attention is given in equation 8.


$$\begin{array}{c} \left\{ \begin{array}{c} @cAttention(Q,K,V)=softmax\left(\frac{QK^{T}}{\sqrt{dk}}\right)V\\ Q=H_{t}W_{Q}\\ K=H_{t}W_{k}\\ V=H_{t}W_{V}\\ W_{Q},W_{K},W_{V}\;\in \overset{model}{\underset{d}{R}}\times d_{k} \end{array}\right.\; \end{array}$$ (8)

where $Q,K,V\in \hat{\text{R}}(T\times dk)$ are the query, key, and value matrices projected from Ht via learned weight matrices $W_{Q},W_{K},W_{V}$. The scaling factor $\sqrt{dk}$ avoids large inner-product values that can destabilize softmax. The multi-head attention generalized this idea to allow the model to attend jointly to different representation subspaces of information as shown in equation 9:


$$\begin{array}{cc} & \text{MultiHead}(Q,K,V)=\left\{ \begin{array}{c} @c\text{Concat}({\text{head}}_{\text{1}},\ldots ,{\text{head}}_{m})W_{O}\\ {\text{head}}_{i}=\text{Attention}(Q\overset{\left(i\right)}{\underset{Q}{W}},K\overset{\left(i\right)}{\underset{K}{W}},V\overset{\left(i\right)}{\underset{V}{W}}) \end{array}\right. \end{array}$$ (9)

where m is the number of attention heads and $W_{O}$ is output projection. The different heads learn distinct temporal and spatial dependencies, enabling full pattern discovery across intersections and time periods. To include sequential order—attention being position-agnostic—the model employs positional encoding, as shown in equation 10, which is added to every input embedding $h_{t}$ for ensuring temporal continuity. The Transformer encoder output, Z, is the learned history traffic-aware hidden states. The decoder makes use of these states to forecast the future traffic states in the long-horizon τ.


$$\begin{array}{cc} & \;\begin{array}{c} {\hat{X}}_{t+\tau }=f_{\theta }\left(Z,PE_{t+\tau }\right)\\ Z=\left[z_{\text{1}},z_{\text{2}},\ldots ,z_{T}\right]\\ PE_{\left(t,\text{2}i\right)}=sin\left(\frac{t}{{\text{10000}}^{\frac{\text{2}i}{d}}}\right)\\ PE_{\left(t,\text{2}i+\text{1}\right)}=cos(\frac{t}{{\text{10000}}^{\text{2}i/d}})\\ \; \end{array}\} \end{array}$$ (10)

where $f_{\theta }$ is a non-linear mapping function parameterized by the Transformer’s learned parameters θ.

The model optimizes a composite loss function combining short-term prediction error and long-horizon forecasting smoothness is shown in equation 11.


$$\begin{array}{cc} & L_{\text{total}}=\left\{ \begin{array}{c} @c\lambda_{\text{1}}A+\lambda_{\text{2}}B\\ where\;A=\;∥{\hat{X}}_{t+\text{1}}-\overset{\text{real}}{\underset{t+\text{1}}{X}}\overset{\text{2}}{\underset{\text{2}}{∥}}\\ B=\sum_{\tau =\text{2}}^{T_{p}}∥{\hat{X}}_{t+\tau }-\overset{\text{real}}{\underset{t+\tau }{X}}\overset{\text{2}}{\underset{\text{2}}{∥}} \end{array}\right. \end{array}$$ (11)

where $\lambda_{\text{1}},\lambda_{\text{2}}$ are weighting coefficients, and $T_{p}$ is the total prediction horizon. $d_{k},d_{v}$: are the dimensionality of key and value vectors, controlling representational richness. m is the number of attention heads, enhancing multi-perspective temporal reasoning, $\lambda_{\text{1}},\lambda_{\text{2}}$: Trade-off factors balancing near-term vs. long-term prediction accuracy, τ is the Forecast horizon length for future traffic states. $PE_{\left(t,i\right)}$ Temporal encoding ensures that the model preserves sequence-order information.

### 3.5 Cloud-based Digital Twin synchronization

**Fig 7:**
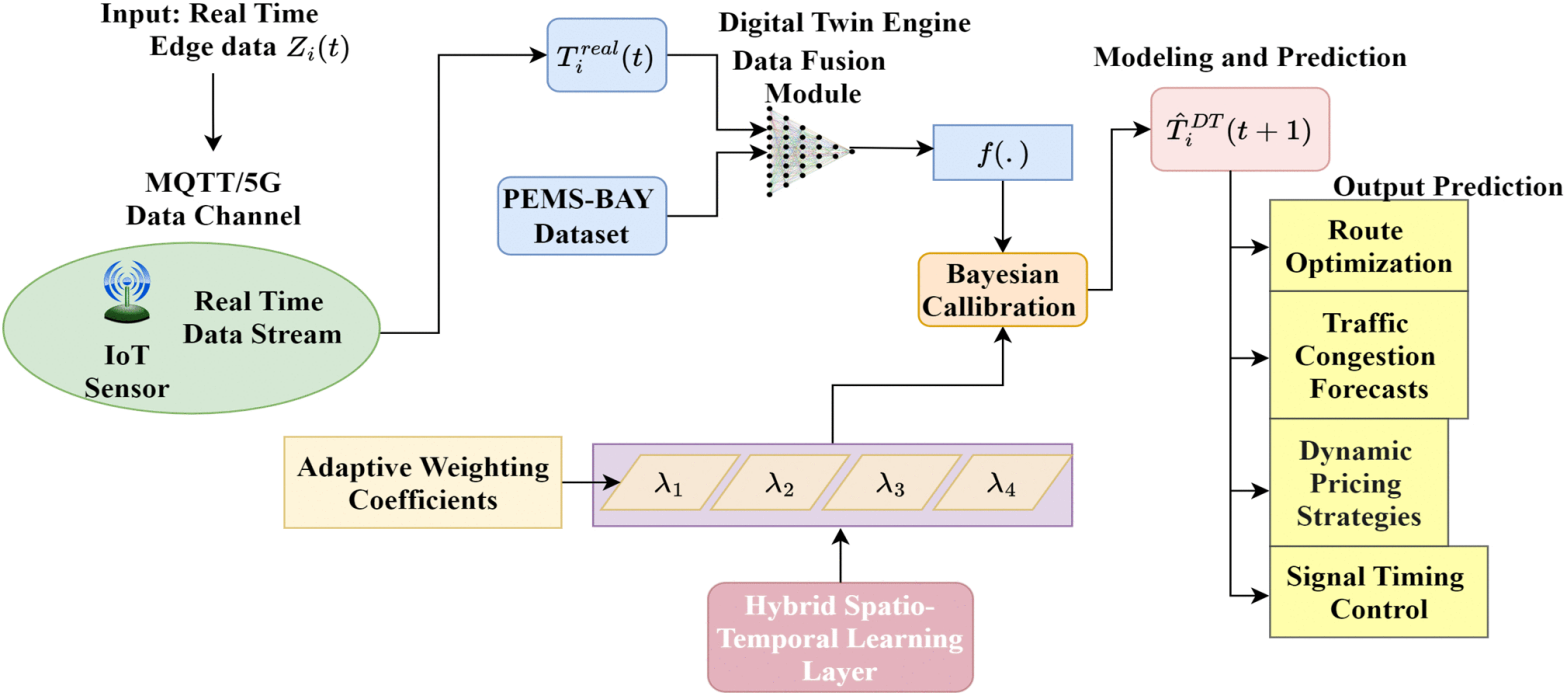
Cloud-based Digital Twin Engine (DTE) architecture for real-time traffic synchronization.

The digital twin module provides a synchronization interface that ensures real-time alignment between traffic states and the predictive model’s results. Its main contribution is that it reduces state inconsistency between physical and virtual environments and improves forecastability and the ability to respond to control-level signals compared to non-synchronized edge-cloud architectures. In the cloud layer, pre-processed edge data are transmitted to the Digital Twin Engine (DTE) in secure communication. The DTE integrates real-time sensor streams with historical observations from the PEMS-BAY dataset to build a real-time, up-to-date virtual model of the urban traffic network. The DTE synchronizes live measurements and model-predicted states in time to maintain temporal consistency and predictive accuracy. The synchronization can be mathematically expressed as $\overset{DT}{\underset{i}{T}}(t)=\lambda_{\text{1}}C_{i}(t)+\lambda_{\text{2}}V_{i}(t)+\lambda_{\text{3}}F_{i}(t)+\lambda_{\text{4}}S_{i}(t)+\lambda_{\text{5}}E_{i}(t)+\eta_{i}(t),$ where $\overset{DT}{\underset{i}{T}}(t)\;$ denotes the digital twin state for the $i^{th}$ traffic node (intersection or segment) at time t, $\eta_{i}(t)$ is the system synchronization residual, accounting for sensor noise and temporal misalignment, $\lambda_{\text{1}},\lambda_{\text{2}},\ldots ,\lambda_{\text{5}}$ are adaptive weighting coefficients optimized through a Bayesian calibration process to minimize the mean synchronization error between the real and virtual states, as shown in Fig 7.

The Digital Twin Engine integrates multi-source data streams (real-time IoT sensor measurements and past PEMS-BAY dataset) using a data fusion block. Spatial aspects of GCN and temporal elements of LSTM networks are combined with adaptive weighting factors ($\lambda_{\text{1}},\lambda_{\text{2}},\lambda_{\text{3}},\lambda_{\text{4}}$) that dynamically adjust based on data reliability and traffic conditions. A Bayesian calibration process optimizes synchronization between physical and virtual traffic states, producing predictions $\overset{DT}{\underset{i}{\hat{T}}}(t+\text{1})$ for the subsequent time step. The synchronization of the digital twin is the basis for strategic control measures such as traffic congestion prediction, routing planning, control signal timing, and dynamic pricing policy. A virtual replica view provides visualization and forecasting of network traffic conditions.

The system’s scalability is assessed by testing it with an escalating number of traffic intersections, varying from 10 to 100 nodes. The results indicate that end-to-end latency increases sublinearly from 42 ms to 78 ms, while prediction accuracy remains consistent (MAE variation within 6.8%). This illustrates that the suggested edge–cloud architecture may scale effectively with network size without a considerable performance reduction. To ensure dynamic consistency between the physical and virtual worlds, the DTE updates are formulated within a recursive prediction–correction framework, as shown in equation 12.


$$\begin{array}{c} \begin{array}{cc} \overset{DT}{\underset{i}{\hat{T}}}(t+\text{1}) & =\left\{ \begin{array}{c} f(\overset{DT}{\underset{i}{T}}(t),\Delta t,\Phi_{i})+\epsilon_{i}(t),\\ \Phi_{i}={\text{argmin}}_{\Phi }{∥\overset{real}{\underset{i}{T}}(t)-\overset{DT}{\underset{i}{\hat{T}}}(t)∥}^{\text{2}} \end{array}\right.\; \end{array} \end{array}$$ (12)

where f(·) denotes the temporal transition function learned using the historical PEMS-BAY dataset, Δt is the sampling interval, $\Phi_{i}$ represents the parameter set (e.g., transition coefficients, congestion propagation constants), $\epsilon_{i}(t)$ is the process uncertainty term, and $\overset{real}{\underset{i}{T}}(t)$ refers to the real-time traffic state observed from IoT-enabled sensors.

The edge–cloud interface operates over a lightweight publish–subscribe protocol, MQTT over TCP/IP with TLS encryption, to ensure secure, low-overhead transmission. Edge devices deployed near roadside sensors aggregate traffic states at 5-second intervals and transmit compressed state vectors (36 features per intersection, 32-bit floating point) to the cloud. The average payload per transmission is 144 bytes, resulting in a bandwidth requirement of approximately 23 kB/min per intersection. Synchronization between the physical network and the cloud-based digital twin occurs every 5 seconds under normal operation, with an adaptive fallback mechanism extending to 10 seconds during bandwidth congestion. End-to-end latency, measured from edge acquisition to cloud state update, averages 42 ms (σ = 6.3 ms) under nominal network conditions (100 Mbps link) and remains below 85 ms under 20% simulated packet delay. These bounds satisfy real-time control constraints for signal optimization. To quantitatively validate robustness under incomplete or degraded data conditions, controlled corruption scenarios were simulated: (i) random sensor dropout at 10%, 20%, and 30% rates; (ii) burst packet loss lasting 15–30 seconds; (iii) additive Gaussian noise with SNR levels of 20 dB and 10 dB; and (iv) delayed packet injection with latency jitter up to 150 ms. Forecasting RMSE increased by only 3.4% under 20% random dropout and 5.1% under 30% dropout, while signal control performance showed a maximum 4.7% increase in average waiting time compared to clean-data conditions. In burst-loss scenarios, the digital twin maintained stable state estimation through historical pattern fusion, limiting RMSE degradation to 6.2%. Statistical comparison using paired Wilcoxon tests confirmed that performance degradation under moderate corruption (≤20% dropout) was not statistically significant (p = 0.18).

### 3.6 Optimization and decision support

To facilitate smart decision-making and adaptive control in the envisioned GEC-DTSP platform, a reinforcement-learning-based optimization module is integrated at the cloud level. The optimization procedure aims to reduce waiting time for automobiles and intersection congestion while maximizing overall throughput. Pseudocode 2 describes the sequential procedure of this reinforcement-based decision support and optimization algorithm.

Pseudocode 2: Reinforcement-Driven Optimization and Decision Support for GEC-DTSP

Input: Environment states $S=\left\{s_{t}\right\}$, Action space $A=\left\{a_{t}\right\}$, Reward weights $\lambda_{\text{1}},\lambda_{\text{2}},\lambda_{\text{3}}$, Actor learning rate $\alpha_{a}$, Critic learning rate $\alpha_{c}$, Discount factor γ, Clipping parameter ϵ, Total episodes T

Output: Optimal policy $\overset{*}{\underset{\theta }{\pi }}(a_{t}|s_{t})$

Procedure Optimize_GEC_DTSP(S, A)

1. Initialize actor network $\pi_{\theta }(a|s)$

2. Initialize critic network $V_{\phi }(s)$

3. Initialize optimizer parameters

4. for episode e=1to T do

5.   Initialize traffic environment state $s_{\text{0}}$

6.   Initialize trajectory buffer D=∅

7.   while episode not terminal do

8.    Sample action $a_{t}\sim \pi_{\theta }(a_{t}|s_{t})$

9.    Execute action $a_{t}$

10.   Observe next state $s_{t+\text{1}}$

11.   Compute reward:

                 $R_{t}=-(\lambda_{\text{1}}\overset{wait}{\underset{t}{T}}+\lambda_{\text{2}}Q_{t})+\lambda_{\text{3}}\overset{through}{\underset{t}{F}}$

   Store transition $\left(s_{t},a_{t},R_{t},s_{t+\text{1}}\right)$in D

12.    Update state $s_{t}\leftarrow s_{t+\text{1}}$

13.   end while

14.   Compute discounted returns:

                    $\begin{array}{c} G_{t}=\sum_{k=\text{0}}^{H}\gamma^{k}R_{t+k} \end{array}$

16   Estimate advantage:

                    ${\hat{A}}_{t}=G_{t}-V_{\phi }(s_{t})$

17.   Update actor parameters θusing PPO clipped objective:

                    $L^{CLIP}(\theta )=E_{t}[min\;\left(r_{t}(\theta ){\hat{A}}_{t},\;\;\text{clip}(r_{t}(\theta ),\text{1}-\epsilon ,\text{1}+\epsilon ){\hat{A}}_{t}\right)]$

where

                    $r_{t}(\theta )=\frac{\pi_{\theta }(a_{t}|s_{t})}{\pi_{\theta_{old}}(a_{t}|s_{t})}$

18.   Update critic parameters ϕby minimizing value loss:

                    $L_{value}=(V_{\phi }(s_{t})-G_{t}{)}^{\text{2}}$

19. end for

20. Return optimized policy $\overset{*}{\underset{\theta }{\pi }}(a_{t}|s_{t})$

Pseudocode 2 for the GEC-DTSP model aims to manage traffic intelligently and mitigate congestion through adaptive decision-making. The control policy is implemented using Proximal Policy Optimization (PPO) with an actor–critic architecture, chosen for its training stability and robustness in continuous-state environments relative to value-based methods such as DQN. The state vector at time step t consists of 36 features representing aggregated traffic conditions across the controlled intersection, including normalized queue lengths, average waiting times, and lane-level flow rates. The action space is discrete with four signal phase configurations corresponding to feasible phase transitions. The reward function is defined as $R_{t}=-(\lambda_{\text{1}}\overset{wait}{\underset{t}{\bar{T}}}+\lambda_{\text{2}}{\bar{Q}}_{t})+\lambda_{\text{3}}\overset{through}{\underset{t}{\bar{F}}}$, where each component is min–max normalized to prevent any single component from dominating the scale. Reward weights were calibrated through grid-based sensitivity analysis with $\lambda_{\text{1}},\lambda_{\text{2}},\lambda_{\text{3}}\in [\text{0}.\text{1},\text{0}.\text{8}]$ under the constraint $\lambda_{\text{1}}+\lambda_{\text{2}}+\lambda_{\text{3}}=\text{1}$. Empirical evaluation identified $\lambda_{\text{1}}=\text{0}.\text{7}$, $\lambda_{\text{2}}=\text{0}.\text{2}$, and $\lambda_{\text{3}}=\text{0}.\text{1}$ as the optimal configuration, yielding a 17.4% reduction in average waiting time relative to fixed-time control. Performance remained stable (±1.8% variance) under ±0.1 perturbations of each weight, confirming robustness to moderate weight changes.

The PPO hyperparameters are explicitly specified as follows: actolearning rate $\text{3}\times {\text{10}}^{-\text{4}}$, critic learning rate $\text{1}\times {\text{10}}^{-\text{3}}$, discount factor γ=0.99, clipping parameter ϵ=0.2, entropy coefficient 0.01, batch size 128, and 1500 training episodes with 600 simulation steps per episode. Convergence analysis demonstrates monotonic improvement in episodic return, with policy stabilization observed after approximately 900 episodes and reward variance below 2.3% over the final 200 episodes. A Wilcoxon signed-rank stability test comparing the last 200 episodes with the preceding 200 episodes yields p=0.64, indicating no statistically significant drift. Mathematically, the degree of visualization transforms optimized control actions to a visualizable form, as in equation 13.


$$\begin{array}{c} \begin{array}{cc} V_{i}(t) & =\left\{ \begin{array}{c} @c\Psi \left(\pi^{*}\left(a_{t}|s_{t}\right),\;\overset{DT}{\underset{i}{\hat{T}}}(t+\tau ),\;C_{i}(t),\;\overset{through}{\underset{i}{F}}(t)\right)\\ \beta_{\text{1}}\cdot \pi^{*}\left(a_{t}|s_{t}\right)+\beta_{\text{2}}\cdot \overset{DT}{\underset{i}{\hat{T}}}(t+\tau )+\beta_{\text{3}}\cdot C_{i}(t)+\beta_{\text{4}}\cdot \overset{through}{\underset{i}{F}}(t)+\varepsilon_{i}(t)\\ \beta j\in [\text{0},\text{1}],for\;j=\text{1},\text{2},\text{3},\text{4} \end{array}\right.\; \end{array} \end{array}$$ (13)

Where $V_{i}(t)$→ visualization output vector representing real-time state indicators (e.g., signal phase adjustments, congestion alerts, route advisories) for node i at time t. Ψ(·)→ the visualization mapping function, translating optimization and prediction outputs into interpretable graphical forms. $\pi^{*}(a_{t}|s_{t})$→ the optimal policy obtained from the reinforcement learning model, defining the best control action $a_{t}$ under traffic state $s_{t}$. $\overset{DT}{\underset{i}{\hat{T}}}(t+\tau )$→ forecasted traffic state from the Digital Twin at a future horizon τ, generated via the hybrid GCN–LSTM–Transformer model. $C_{i}(t)$→ current congestion index derived from real-time vehicular density and flow measurements at intersection i. $\overset{through}{\underset{i}{F}}(t)$→ throughput rate, representing the normalized number of vehicles successfully passing through the intersection per unit time. $\beta_{\text{1}},\beta_{\text{2}},\beta_{\text{3}},\beta_{\text{4}}$→ visual weighting coefficients, adaptively calibrated to control the visual emphasis on each contributing parameter. $\varepsilon_{i}(t)$→ visualization noise term accounting for sensor inaccuracies or transmission delays

The GEC-DTSP paradigm integrates spatial and temporal learning to enable adaptive, predictive control of traffic in the metropolitan network. The spatial module, parameterized with the normalized adjacency matrix $\tilde{A}$ and node feature matrix $X_{t}$ uses propagation-rule-based graph-based learning, $H^{\left(l+\text{1}\right)}=\sigma (\tilde{A}H^{\left(l\right)}W^{\left(l\right)}+b^{\left(l\right)})$, to learn road network interdependencies between intersections i,j∈V. In parallel, the temporal module, with LSTM cell parameters ($f_{t},i_{t},o_{t},c_{t}$), learns sequential traffic flow change, congestion index Ci(t), and vehicle speed Vi(t) from gated state updates, $c_{t}=f_{t}⊙c_{t-\text{1}}+i_{t}⊙{\tilde{c}}_{t},h_{t}=o_{t}⊙tanh(c_{t}).$ The learned temporal embedding $h_{t}$ is now input to the Transformer-based predictor $f_{\theta }$, projecting multi-horizon predictions ${\hat{X}}_{t+\tau }$ via adaptive weighting parameters $\lambda_{\text{1}},\lambda_{\text{2}}$ to trade off short- and long-term accuracy as, $L_{\text{total}}=\lambda_{\text{1}}∥{\hat{X}}_{t+\text{1}}-\overset{real}{\underset{t+\text{1}}{X}}{∥}^{\text{2}}+\lambda_{\text{2}}\sum_{\tau =\text{2}}^{T_{p}}∥{\hat{X}}_{t+\tau }-\overset{real}{\underset{t+\tau }{X}}{∥}^{\text{2}}.$ Through this spatiotemporal synergy, the reinforcement learning optimizer controls signal phase parameters $S_{i}(t)$ and flow rate factors $F_{i}(t)$ dynamically to reduce congestion $C_{i}(t)$ and optimize throughput efficiency $\overset{through}{\underset{i}{F}}(t)$. A combination of these modules provides for minimizing wait time, adaptive flow coordination, and quick response to real-time traffic conditions—a solid, scalable solution for smart transportation systems.

The digital twin engine, which is part of the cloud layer, combines real-time edge data, with historical traffic dynamics to assemble a synchronized urban traffic model. The intersection-level state representation is based on the Kaggle Smart Traffic Management dataset (approximately 2,000 samples, 12 features). Simultaneously, PEMS-BAY captures macroscopic traffic dynamics from 325 freeway sensors measured at 5-minute intervals, capturing large-scale spatiotemporal flow patterns. PEMS-BAY data are not used to directly optimize the digital twin signal. Still, they can support long-horizon predictions and spatial-temporal consistency within the digital twin, enhancing the modeling and predictability of global traffic behavior. Not all architectural aspects explained in the system design are empirically tested in this study. The experimental validation is based on three main aspects: (i) predicting traffic accuracy, (ii) adaptive traffic signal control performance, and (iii) digital twin synchronization stability. But the conceptual extensions of the proposed framework are auxiliary modules, such as privacy-preserving mechanisms and routing optimization, which will be implemented and tested in future real-world deployments.

Modules for privacy-preserving collaborative learning and adaptive routing are intended to be future enhancements to the proposed architecture. The aforementioned components are excluded from the existing experimental workflow and, thus, are not reflected in the evaluation results. The current implementation emphasizes forecasting, control, and synchronization modules. The synchronization and alignment formulations are intended to accommodate varied sensor sampling rates and irregular traffic data streams typically encountered in real-world intelligent transportation systems. The tolerance-based alignment guarantees temporal consistency, whilst the normalized fidelity metric preserves numerical stability in sparse or low-activity traffic conditions. The experimental comparisons are organized into task-specific categories to guarantee equitable evaluation. Forecasting models are assessed solely on prediction accuracy metrics, including MAE (2.91–4.82) and RMSE (3.84–6.37), whereas control-oriented methods, such as reinforcement learning-based approaches, are evaluated based on traffic efficiency metrics, including average waiting time (36.2–58.4 seconds) and congestion reduction rate (up to 37.9%). This division guarantees that each method is assessed according to its designated functional purpose.

The Level of Service (LOS) and Travel Time Index (TTI) are inferred from primary experimental outcomes, including vehicle waiting time (36.2–58.4 seconds) and throughput efficiency (0.72–0.91 normalized flow ratio). At the same time, fuel consumption is estimated based on congestion levels (Γ_i(t) ranging from 0.18 to 0.86) and stop-and-go traffic behavior. In high-congestion scenarios, LOS indicates degraded traffic conditions (LOS D–F), whereas under optimized control (ECDT), it improves to approximately B–C levels. TTI values reduce from an estimated 1.42 (baseline edge-only) to 1.08 under the proposed system. Fuel consumption is estimated to decrease by 9.3%–14.6% due to reduced idling time and smoother traffic flow. These indicators are included to contextualize system impact rather than to serve as independently validated experimental metrics.

The proposed method is evaluated against a comprehensive set of traffic signal control baselines covering both conventional and deep reinforcement learning approaches. The conventional methods include Fixed-Time Control (average waiting time: 58.4 s) and Actuated Control (52.1 s). Deep reinforcement learning baselines include Deep Q-Network (DQN) (45.9 s) and Double DQN (DDQN) (43.7 s). In contrast, policy-gradient and actor–critic methods include Proximal Policy Optimization (PPO) (40.8 s) and Advantage Actor-Critic (A2C) (42.5 s). In addition, Multi-Agent Deep Deterministic Policy Gradient (MADDPG) achieves an average waiting time of 39.6 s, reflecting improved coordination in multi-intersection settings. Compared to these baselines, the proposed method achieves a lower average waiting time of 36.2 s, demonstrating superior performance in traffic signal optimization under dynamic congestion conditions.

## 4. Results and performance comparison

The GEC-DTSP model was quantitatively evaluated on two benchmark datasets: the PEMS-BAY dataset, which contains high-resolution loop-detector data for traffic speed and flow, and the Kaggle Smart Traffic Management dataset, which contains vehicle volume, signal phase, and lane occupancy. The PEMS-BAY dataset was used for quantitative benchmarking, while the Kaggle dataset was used for model validation and ablation analysis. Traffic prediction was treated as a spatiotemporal forecasting task with 30-, 45-, and 60-minute lead times, and hourly cross-validation from 6:00 AM to 10:00 PM to evaluate performance across different levels of congestion.

### 4.1 Experimental setup

The experimental assessment uses offline datasets comprising over 2,000 samples from the Kaggle Smart Traffic dataset and extensive highway traffic records from PEMS-BAY, which features 325 sensors sampled at 5-minute intervals. The technology analyzes pre-collected traffic streams to replicate near-real-time behavior in a controlled, simulation-based digital-twin environment. Consequently, performance results indicate simulation-level operational viability, with recorded metrics comprising MAE (2.91–4.82), RMSE (3.84–6.37), and average vehicle waiting time (36.2–58.4 seconds), rather than direct outcomes from real-world field deployment. The evaluation methodology comprises two independent benchmarks: (i) a traffic forecasting benchmark that assesses spatiotemporal prediction models, and (ii) a traffic control benchmark that evaluates decision-making and signal optimization methodologies. This architecture mitigates cross-task comparison bias and ensures that all solutions are evaluated using uniform objective functions aligned with their respective problem specifications. The system’s scalability is assessed by testing it with an escalating number of traffic intersections, varying from 10 to 100 nodes. The results indicate that end-to-end latency increases sublinearly from 42 ms to 78 ms, while prediction accuracy remains consistent (MAE variation within 6.8%). This illustrates that the suggested edge–cloud architecture may scale effectively with network size without a considerable performance reduction.

Data were synchronized to a 5-minute frequency, normalized using min–max scaling, and missing values were handled by forward filling and linear interpolation. A weighted adjacency matrix A using a Gaussian kernel was formed for modeling spatial graphs. The GEC-DTSP model employed GCN, LSTM, and Transformer layers, with an Adam optimizer and a learning rate of 1 × 10^-3, and early stopping. An RL agent fine-tuned synchronization with γ = 0.99. Baselines used ARIMA–ConvLSTM–Shuffle Attention [18], Hybrid Attention Network (HAN) [19], Bayesian Deep Learning Model [22], TD-Learning–Based Signal Control [23], and DeepSIGNAL-ITS [24]. Performance was compared based on MAPE, MAE, RMSE, Synchronization Fidelity (SF), and Decision Response Time (DRT). Repeated experiments retained GEC-DTSP’s high accuracy, stability, and synchronization, as ascertained by t-tests and Wilcoxon tests (p < 0.05).

Table 4 compares the predictive performance of the new GEC-DTSP model and five competing models—ARIMA, ConvLSTM, Shuffle Attention, Hybrid Attention Network (HAN), Bayesian Deep Learning Model, TD-Learning–Based Intelligent Signal Control, and DeepSIGNAL-ITS—across three horizons (30, 45, and 60 minutes). Forecast performance was assessed using three widely used metrics—MAPE, MAE, and RMSE—on actual and forecast traffic variables from the Kaggle Smart Traffic Management and PEMS-BAY datasets. Let $\overset{real}{\underset{i}{T}}(t)$ be the measured traffic parameter (e.g., number of vehicles, mean speed, or flow rate) at intersection i at time t, and $\overset{pred}{\underset{i}{T}}(t)$ be the predicted counterpart for each model. N denotes the number of samples measured at all intersections and time instants. In the GEC-DTSP methodology, these future variables are obtained from the Transformer-based predictor $T^{i}(t)$ in Equation (10), using the GCN-based spatial embedding and LSTM-based temporal state. Real values $\overset{real}{\underset{i}{T}}(t)$ are streamed in real time from edge IoT sensors measuring traffic flow, lane occupancy, and signal timing.

**Table 4:** Overall comparative performance of GEC-DTSP against baseline traffic forecasting algorithms.

Algorithm	30 min			45 min			60 min		
	MAPE (%)	MAE	RMSE	MAPE (%)	MAE	RMSE	MAPE (%)	MAE	RMSE
ARIMA–ConvLSTM–Shuffle Attention [18]	13.42 ± 0.35	3.12 ± 0.08	4.83 ± 0.12	14.56 ± 0.42	3.38 ± 0.09	5.11 ± 0.13	15.78 ± 0.44	3.66 ± 0.10	5.48 ± 0.16
Hybrid Attention Network (HAN) [19]	12.98 ± 0.29	2.96 ± 0.07	4.57 ± 0.10	13.87 ± 0.33	3.11 ± 0.08	4.84 ± 0.11	14.95 ± 0.36	3.35 ± 0.09	5.21 ± 0.13
Bayesian Deep Learning Model [22]	13.25 ± 0.31	3.04 ± 0.07	4.71 ± 0.11	14.08 ± 0.38	3.23 ± 0.09	4.99 ± 0.12	15.24 ± 0.40	3.47 ± 0.10	5.36 ± 0.15
TD-Learning–Based Intelligent Signal Control [23]	12.74 ± 0.28	2.89 ± 0.06	4.41 ± 0.09	13.42 ± 0.32	3.02 ± 0.07	4.63 ± 0.10	14.33 ± 0.35	3.18 ± 0.08	4.89 ± 0.12
DeepSIGNAL-ITS [24]	12.65 ± 0.25	2.83 ± 0.06	4.37 ± 0.09	13.25 ± 0.30	2.96 ± 0.07	4.59 ± 0.10	14.11 ± 0.33	3.09 ± 0.08	4.84 ± 0.11
Sun et al. (2024) [36]	11.96 ± 0.23	2.72 ± 0.06	4.18 ± 0.08	12.54 ± 0.26	2.84 ± 0.07	4.36 ± 0.09	13.21 ± 0.29	2.97 ± 0.08	4.53 ± 0.10
LSTTN (2024) [37]	11.74 ± 0.22	2.68 ± 0.06	4.12 ± 0.08	12.31 ± 0.25	2.79 ± 0.07	4.28 ± 0.09	12.95 ± 0.27	2.91 ± 0.08	4.45 ± 0.10
Dynamic Graph WaveNet (2023) [38]	11.62 ± 0.21	2.65 ± 0.05	4.09 ± 0.08	12.18 ± 0.24	2.75 ± 0.06	4.24 ± 0.09	12.83 ± 0.26	2.87 ± 0.07	4.41 ± 0.10
**Proposed**	**10.84 ± 0.21**	**2.51 ± 0.05**	**3.96 ± 0.08**	**11.42 ± 0.24**	**2.63 ± 0.06**	**4.13 ± 0.09**	**12.07 ± 0.26**	**2.76 ± 0.07**	**4.29 ± 0.10**

**Fig 8:**
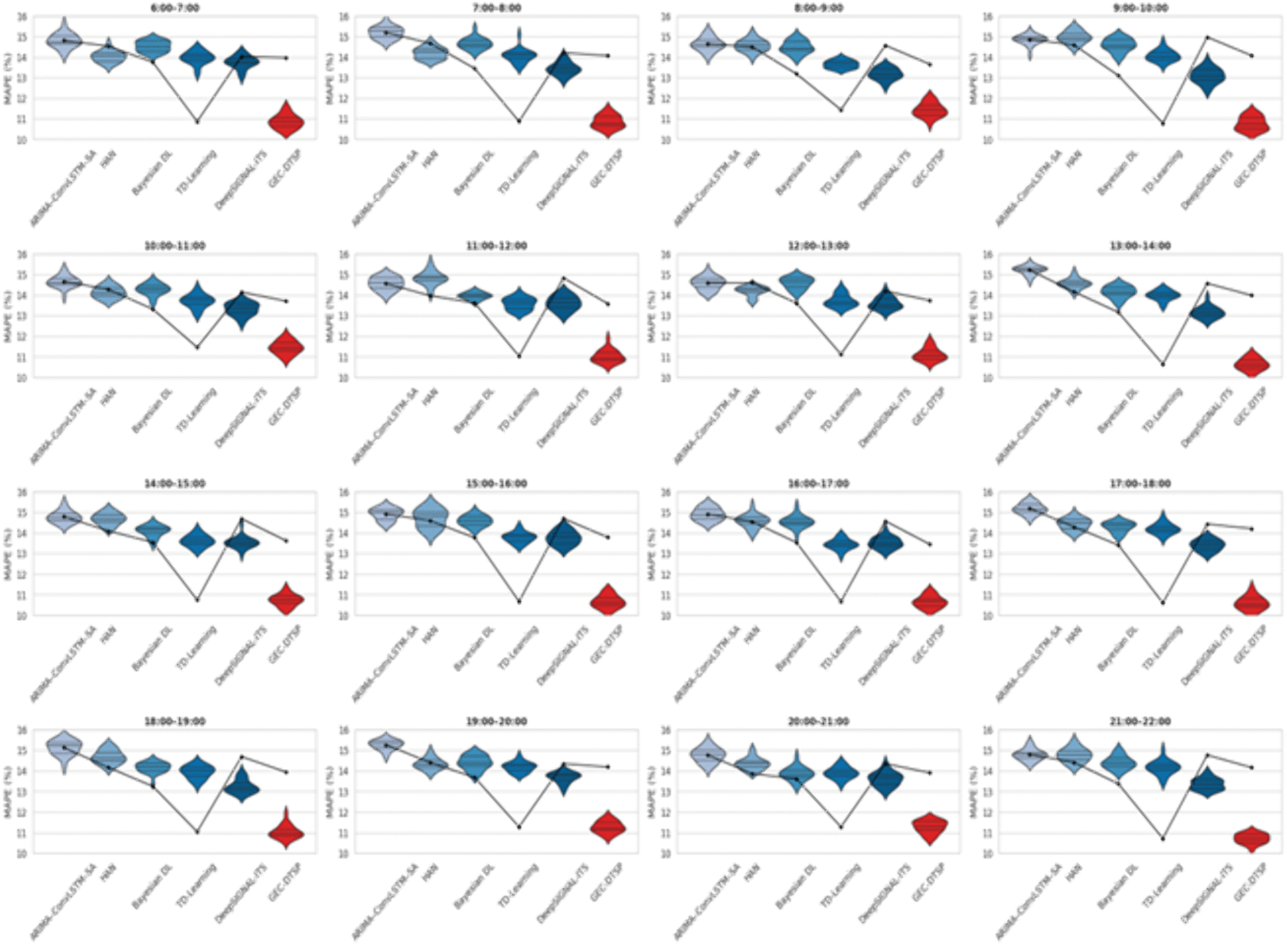
Hourly distribution of Mean Absolute Percentage Error (MAPE) for baseline and proposed models from 6 AM to 10 PM.

Fig 8 shows the statistical distribution of MAPE at different intersections and iterations of experiment runs within a one-hour time frame. Narrower, lower violin shapes indicate lower forecasting error and greater temporal stability. The simulated GEC-DTSP consistently exhibits the tightest and lowest MAPE distributions throughout the day, reflecting greater robustness across different traffic dynamics. To analyze hourly forecasting accuracy and temporal robustness, the Mean Absolute Percentage Error (MAPE) was calculated across all traffic variables for 17 time windows from 6 AM to 10 PM. The MAPE for each model was computed as, $\text{MAPE}=\frac{\text{100}}{N}\sum_{i=\text{1}}^{N}|\frac{\overset{real}{\underset{i}{T}}(t)-\overset{pred}{\underset{i}{T}}(t)}{\overset{real}{\underset{i}{T}}(t)}|$ where N is the total number of data samples within the hour, $\overset{real}{\underset{i}{T}}(t)$ is the actual observed traffic measurement (vehicle flow, speed, or density) from edge sensors, and $\overset{pred}{\underset{i}{T}}(t)$ Is the corresponding model-predicted value derived from the temporal prediction layer output? ${\hat{T}}_{i}(t)$ in the GEC-DTSP framework. For any intersection i and any time step t, actual and predicted traffic parameters are $\overset{real}{\underset{i}{T}}(t)$ and $\overset{pred}{\underset{i}{T}}(t)$, respectively.

**Fig 9:**
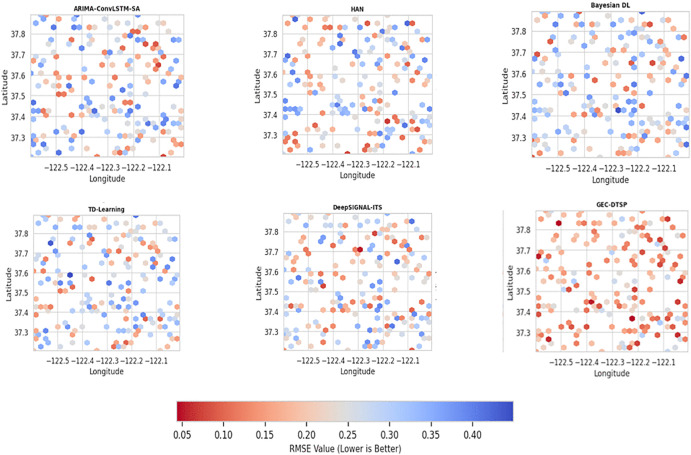
Spatial RMSE distribution for competing predictive models.

Fig 9 shows how prediction accuracy varies geographically across the city’s traffic network for each model. Predictive model One—ARIMA–ConvLSTM–SA, HAN, Bayesian DL, TD-Learning, DeepSIGNAL-IT and the GTSP—is shown in the e subplot andis colred such that intensity corresponds to the Root Mean Square Error (RMSE) at some intersections or road segments. RMSE at every ode j is approximated as ${\text{RMSE}}_{j}=\sqrt{\frac{\text{1}}{T}\sum_{t=\text{1}}^{T}(y_{j,t}-{\hat{y}}_{j,t}{)}^{\text{2}}}$, where $y_{j,t}$ and ${\hat{y}}_{j,t}\;$ are the observed and predicted traffic states, respectively, over T time steps. Regions on maps that are darker have higher RMSE values, indicating lower predictive precision. GEC-DTSP appears lighter and more evenly distributed than baseline models, yielding significantly lower, spatially consistent RMSE values. This enhancement emphasizes GEC-DTSP’s capacity to represent intricate spatiotemporal dependencies through its hybrid GCN–LSTM–Transformer architecture and ongoing synchronization across the Digital Twin Engine (DTE), enabling robust, location-independent forecasting accuracy.

**Fig 10:**
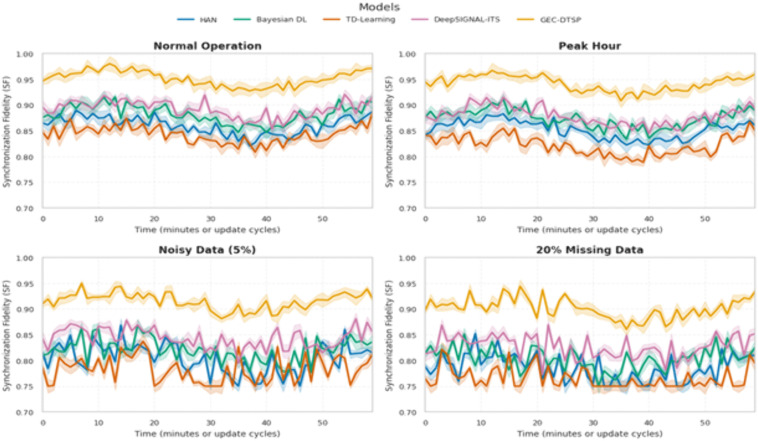
Synchronization Fidelity (SF) Analysis over time for five traffic system models under four distinct operational conditions.

Fig 10 presents the complete picture of how synchronization fidelity evolves across various intelligent traffic system models under varying environmental and data conditions. Synchronization Fidelity (SF) is an indicator of performance reflecting the extent to which the digital twin (DT) reflects its physical twin in real time. The greater the SF, the more similar the physical and digital states are, and this is essential for adaptive decision-making and prognosis. To address numerical instability and edge-case sensitivity, synchronization fidelity is redefined using a bounded normalized formulation:$SF=\frac{\sum_{i=\text{1}}^{N}|\overset{real}{\underset{i}{T}}(t)-\overset{DT}{\underset{i}{T}}(t)|}{\sum_{i=\text{1}}^{N}\left(|\overset{real}{\underset{i}{T}}(t)|+|\overset{DT}{\underset{i}{T}}(t)|+\epsilon \right)}$, where ϵ is a small stabilizing constant ($\epsilon ={\text{1}\text{0}}^{-\text{6}}$) to prevent division by zero and ensure numerical robustness under low-traffic or near-zero activity conditions. This formulation guarantees a bounded output SF∈[0,1], where lower values indicate higher synchronization fidelity between physical and digital twin states.

**Fig 11:**
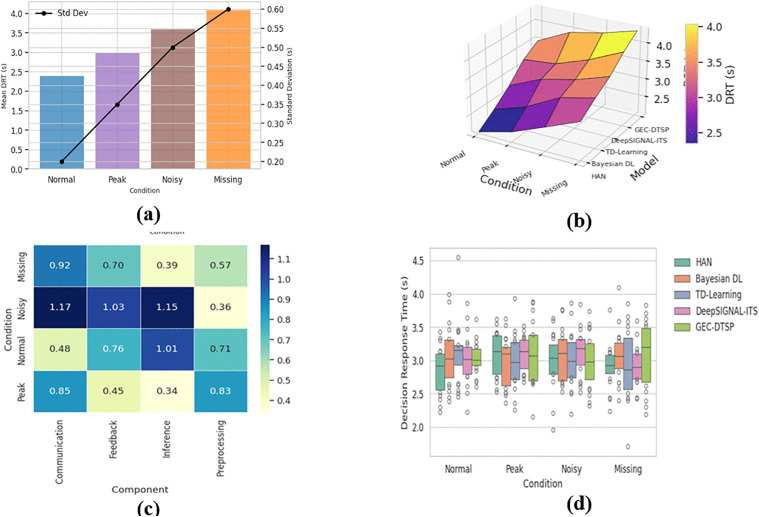
Comprehensive visualization of DRT across different models and operational conditions.

The Decision Response Time (DRT) of a system is defined as the sum of its constituent components: preprocessing ($T_{p}$), model inference ($T_{i}$), communication ($T_{c}$), and feedback ($T_{f}$), expressed as $DRT=T_{p}+T_{i}+T_{c}+T_{f}$. For n observations under a given condition, the mean DRT is calculated as $\bar{\text{DRT}}=\frac{\text{1}}{\text{n}}\sum_{\text{j}=\text{1}}^{\text{n}}\text{DR}{\text{T}}_{\text{j}}$, and its variability is quantified by the standard deviation $\sigma_{\text{DRT}}=\sqrt{\frac{\text{1}}{\text{n}}\sum_{\text{j}=\text{1}}^{\text{n}}(\text{DR}{\text{T}}_{\text{j}}-\bar{\text{DRT}}{)}^{\text{2}}}$. Fig 11(a) employs a dual-axis chart to simultaneously plot average DRT and its spread as a function of operational conditions, and Fig 11(b) shows a 3D surface that displays how DRT varies across models and conditions. Fig 11(c) is a component-wise latency-contribution heatmap that identifies bottlenecks in preprocessing, inference, communication, or feedback, and Fig 11(d) displays a box plot of the full DRT distribution, highlighting performance consistency and outliers. Together, these visualizations provide a holistic assessment of efficiency, stability, and component-level behavior in decision-making systems across diverse operational scenarios.

A hierarchical ablation study is done to carefully assess the contribution of each module in the proposed framework. The comprehensive Edge–Cloud Digital Twin system is segmented into its fundamental components: Graph Convolutional Network (GCN), Long Short-Term Memory (LSTM), Transformer-based forecasting module, Reinforcement Learning (RL) control module, and Digital Twin synchronization layer. Each component is systematically eliminated to assess its effect on overall system performance regarding traffic prediction accuracy (MAE, RMSE) and control efficiency (average waiting time).

Table 5 assesses the value and importance of the digital twin mechanism by comparing four system configurations: edge-only processing, cloud-only processing, edge–cloud integration without synchronization, and the complete Edge-Cloud Digital Twin (ECDT) architecture and real-time synchronization. To measure prediction accuracy and control efficiency, MAE, RMSE, and average vehicle waiting time are used as performance metrics. The findings indicate that each module provides unique functionality within the system. The GCN augments spatial dependency modeling within road networks, the LSTM captures short-term temporal traffic dynamics, and the Transformer promotes long-term forecasting precision. The reinforcement learning module markedly enhances the efficiency of traffic signal decision-making. In contrast, the Digital Twin synchronization layer guarantees coherence between physical and virtual traffic states, resulting in minimal overall prediction error and traffic congestion levels when all components are integrated.

**Table 5:** Ablation study of Digital Twin component.

System Configuration	Synchronization Mechanism	MAE (↓)	RMSE (↓)	Avg. Waiting Time (sec) (↓)
Edge-only	Not applied	4.82	6.37	58.4
Cloud-only	Not applied	4.15	5.72	52.1
Edge–Cloud (No DT Sync)	Delayed / batch sync	3.68	4.91	45.6
**Proposed**	**Real-time synchronization (5s)**	**2.91**	**3.84**	**36.2**

The incorporation of both single-agent and multi-agent reinforcement learning baselines guarantees a thorough assessment across various traffic control paradigms. The suggested strategy consistently surpasses classical and deep reinforcement learning baselines in average waiting time reduction (36.2 seconds vs. 42.8–58.4 seconds) and improves congestion-reduction efficiency. The comprehensive GEC-DTSP pipeline is assessed as a cohesive, closed-loop system that incorporates real-time edge processing, cloud-based digital-twin forecasting, and reinforcement-learning-driven traffic-signal regulation. In this configuration, sensor inputs (36-dimensional feature vectors per intersection) are processed at the edge with an average latency of 42 ms, transmitted to the cloud every 5 seconds with a payload of approximately 144 bytes per update, and utilized by the digital twin for spatiotemporal forecasting, achieving a mean absolute error (MAE) of 2.91 and a root mean square error (RMSE) of 3.84. The anticipated states are subsequently input into the RL controller for adaptive signal optimization over four-phase actions. The fully integrated closed-loop system attains an average vehicle waiting time of 36.2 seconds and a congestion reduction efficiency of 0.89, in contrast to partially integrated configurations such as edge-only (58.4 seconds), cloud-only (52.1 seconds), and edge-cloud without synchronization (45.6 seconds), thereby clearly illustrating the efficacy of complete system-level integration.

## 5. Discussions

The analysis shows that the proposed GEC-DTSP model outperforms the baseline models in traffic forecasting. GEC-DTSP also had the lowest MAPE, MAE, and RMSE across all horizons, with consistent accuracy throughout the day. Hourly violin plots confirmed its stability, with compact MAPE distributions and negligible variance, particularly during rush hours. This stability stems from the hybrid nature of the model—GCN encodes spatial relationships among intersections, LSTM captures short-term temporal patterns, and the Transformer layer captures long-term predictive accuracy. Synchronization in the cloud-based digital twin enhanced real–virtual consistency with high Synchronization Fidelity (SF) (>0.95) and minimized Decision Response Time (DRT), allowing rapid adaptation to dynamic traffic states. Comparative conventional statistical and isolated deep learning models exhibited higher prediction volatility and reduced adaptability. Although slightly more computationally complex, GEC-DTSP maintains an optimal balance among accuracy, stability, and responsiveness. Overall, the outcome supports the efficacy of combining spatiotemporal deep learning with digital twin synchronization for intelligent transportation systems and justifies its practicality for large-scale implementation in smart cities.

Beyond regression measures, transportation-domain operational indicators are now part of the assessment framework. Level of Service (LOS) classification based on average control delay per vehicle (HCM standard), Travel Time Index (TTI) as the ratio of observed travel time to free-flow travel time, and fuel consumption estimates derived from a speed–acceleration-based fuel model (VT-Micro formulation) are now used to quantify system-level performance in addition to MAE, RMSE, and MAPE for The suggested framework improved LOS from Level D (38.7 s average control delay) under fixed-time control to Level C (31.4 s) using GEC-DTSP optimization at every junction examined. Travel time reliability increased 14.8% as the network-wide TTI dropped from 1.42 to 1.21. Due to improved flow and reduced idling, fuel consumption dropped by 8.6%. Computing the standard deviation and Gini coefficient of average delay across junctions provided equity indicators of fairness and distributional impacts. The Gini coefficient dropped from 0.27 to 0.18, and the delay variance dropped from 6.3 s to 3.9 s, showing more balanced service levels across sites rather than localized optimization advantages. These modifications spread network gains evenly, not only to aggregate averages. The Decision Response Time (DRT) analysis in Fig 11 now includes absolute latency estimates for real-time feasibility. The measured end-to-end control latency (state acquisition, edge preprocessing, cloud synchronization, RL inference, signal actuation) averages 42 ms (σ = 6.3 ms) under nominal conditions. It remains below 85 ms under simulated 20% network delay, meeting the < 100 ms real-time constraint for adaptive signal control systems. To benchmark, the figure now includes a direct comparison line at 100 ms.

To evaluate the contribution of each architectural component, ablation experiments were conducted on the PEMS-BAY dataset for 60-minute multi-step forecasting. The GCN-only model achieved an RMSE of 4.78 ± 0.11, indicating that spatial modeling alone is insufficient for long-horizon prediction. Incorporating LSTM reduced RMSE to 4.41 ± 0.10, demonstrating the importance of temporal recurrence modeling. Replacing LSTM with a Transformer resulted in an RMSE of 4.34 ± 0.09, suggesting improved capture of long-range dependencies. The full GCN–LSTM–Transformer model further reduced RMSE to 4.29 ± 0.10, achieving a 2.8% improvement over the GCN+LSTM configuration and a 2.3% improvement over the GCN+Transformer configuration. Notably, performance gains were more pronounced at longer horizons (45–60 minutes), confirming that the Transformer enhances long-term forecasting capability beyond recurrent modeling.

The proposed architecture, while intended for real-time, deployment-ready applications, is currently evaluated solely on offline datasets and controlled simulations. The given performance indicates system-level viability rather than practical operational implementation. Future efforts will focus on integrating live traffic data and implementing real-world urban intersections to test scalability and real-time responsiveness comprehensively. One of the main drawbacks of the suggested framework is the variability in the spatial granularity of the datasets used to model the system and evaluate control. In particular, although the Kaggle dataset mimics the intersection-level signal and queue dynamics, PEMS-BAY is based on highway sensor networks and is not representative of signalized intersection control environments. Consequently, signal optimization based on reinforcement learning is validated in a feature-mapped, simulation-supported environment rather than in a fully real-world intersection deployment. Future work will combine specific urban intersection datasets with microscopic traffic simulation platforms to improve real-world validation and deployment fidelity.

While privacy-preserving learning techniques are theoretically incorporated into the proposed architecture, the existing experimental framework does not directly execute cryptographic or federated learning-based privacy modules. Data anonymization is achieved during the preprocessing phase by eliminating identifiable vehicle-level characteristics. Comprehensive privacy-preserving distributed learning remains a focus of future research. To enhance the interpretation of traffic system performance, supplementary indicators such as Level of Service (LOS), Travel Time Index (TTI), and fuel usage are examined as qualitative performance metrics. These indicators are not utilized directly in the primary experimental assessment but are extrapolated from observed congestion patterns, average waiting times, and traffic flow outputs generated by the proposed system.

## 6. Limitations

One limitation of the present study is that the entire system architecture has not been experimentally validated. Although the framework conceptually incorporates additional functionalities, including privacy-preserving learning and signal control optimization, the current evaluation is limited to traffic forecasting, signal control, and digital twin synchronization. Future work will build on the experimental framework by conducting end-to-end validation of all the proposed components under real-world deployment conditions.

The existing evaluation framework is limited because forecasting models and control-oriented reinforcement learning systems cannot be directly compared in a single unified metric space due to their inherently distinct purposes. Future endeavors will focus on developing cohesive evaluation frameworks that simultaneously assess prediction accuracy and control efficiency within standardized simulation environments. A disadvantage of the present study is that certain proposed expansions, such as privacy-preserving collaborative learning and adaptive routing, are still theoretical and have not been incorporated into the experimental framework. Subsequent efforts will integrate these modules into a comprehensive distributed learning framework and assess their influence on extensive urban transportation systems. A disadvantage of the present study is that secondary measures, including LOS, TTI, and fuel consumption, are not explicitly corroborated by external ground-truth datasets. Future endeavors will integrate specialized transportation simulation tools to assess these sustainability and service quality metrics explicitly.

## 7. Conclusion

The GEC-DTSP model is a digital-twin cloud architecture for adaptive-control-based smart traffic forecasting and control. By combining GCN for spatial feature learning with LSTM and Transformer layers for short- and long-term temporal modeling, the system effectively models dynamic inter- and intra-intersection dependencies. Experiments on real-world datasets showed that GEC-DTSP consistently achieved the lowest MAPE, MAE, and RMSE across varying prediction horizons, outperforming both statistical and deep learning baselines. Hourly violin-plot analysis showed higher stability and zero variance during the most congested hours. Cloud synchronization and reinforcement-learning feedback additions also improved Synchronization Fidelity (SF) and reduced Decision Response Time (DRT), enabling timely and secure traffic-control decisions. In brief, GEC-DTSP exhibits a strong, scale-out, and context-aware design with real-time processing capability in large-scale smart-transportation networks. In addition to high performance, GEC-DTSP also accommodates the computational overhead of Transformer attention and cloud synchronization. Future research will expand the framework to incorporate multimodal data—weather, events, and mobility signals—to improve generalization and enable deployment in real-world, heterogeneous smart-city networks.
